# A Global Review of the Pests and Diseases of Stingless Bees

**DOI:** 10.3390/insects17060619

**Published:** 2026-06-11

**Authors:** Robert McDougall, Robert Spooner-Hart, James Cook

**Affiliations:** Hawkesbury Institute for the Environment, Western Sydney University, Richmond, NSW 2753, Australia; r.spooner-hart@westernsydney.edu.au (R.S.-H.); james.cook@westernsydney.edu.au (J.C.)

**Keywords:** meliponiculture, pests, disease, viruses, bacteria, fungi, parasites

## Abstract

Managed pollinators are essential to agricultural productivity, but there are concerns that overreliance on western honey bees (*Apis mellifera*) places this productivity at risk. Stingless bees (Apidae; tribe Meliponini) can supplement or replace honey bees as pollinators in some warm climate crops, but information is limited regarding the pests and diseases that they face. This paper reviews the existing research literature on the subject. We found 48 studies on arthropod pests and 28 on microbial diseases of stingless bees. There has been a relatively high research interest in mites (Acari) and small hive beetles (*Aethina tumida*); however, neither of these organisms are major pests of stingless bees. This high research interest likely results from their impact on honey bees and their novelty as newly arrived invasive species in many regions. Cleptoparasitic bees have also been highly researched. The most widely researched diseases were viruses. However, while many widespread honey bee viruses have been found in stingless bees, few studies examined whether these produced symptoms in stingless bees. For many suspected pests and pathogens, there is uncertainty regarding whether their relationship with hive collapse is causal or merely correlative. We conclude that pests and diseases of stingless bees are very under-researched compared to those of honey bees. This knowledge gap will need to be addressed if stingless bees are to serve an expanded role as managed agricultural pollinators.

## Simple Summary

Farmers of many pollinator-dependent crops utilize managed pollinators. However, globally, there is heavy reliance on a single species, the western honey bee (*Apis mellifera*), placing crop productivity at risk from shocks that impact honey bees. Stingless bees (Apidae; tribe Meliponini) are a highly diverse group of eusocial bees that share many of the traits that make honey bees ideal managed pollinators and could serve as a valuable substitute or additional pollinator for some warm-climate crops. However, information is limited on the pests and diseases that stingless bees face. This paper is a comprehensive review of published stingless bee pest and disease research; we found 48 studies examining arthropod pests and 28 examining diseases. There has been high research interest in resource stealing by other bees, in mites (Acari), and in the small hive beetle (*Aethina tumida*), but these are not necessarily the most widespread or damaging pests. Indeed, much of the research reviewed shows uncertainty about whether potential pests actually cause hive collapse in stingless bees or simply co-occur with unhealthy hives. While many viruses of honey bees can be found in stingless bees, most research results failed to determine if these viruses cause disease symptoms in stingless bees. This review concluded that there is a substantial knowledge gap regarding the harms that pests and pathogens may cause to stingless bees.

## 1. Introduction

Pollinators improve the productivity of around 75% of global crop species [1], and their importance is growing as the proportion of land dedicated to pollinator-dependent crops increases [2]. Managed pollinators play an important role in ensuring that crops receive necessary pollination services, with the most widely used managed pollinator being the western honey bee (*Apis mellifera*), which has been introduced to all inhabited continents [3]. However, there are growing concerns that overreliance on a single pollinator species leaves global agriculture vulnerable to shocks that impact honey bees, such as from pests, diseases and climate change [4]. Thus, diversifying the pollinators on which agricultural systems rely should improve their resilience.

While a number of alternative pollinators currently see some level of human management, including the Asian honey bee (*Apis cerana*), bumble bees, solitary bees and flies [3], the group with the most potential to supplement or replace honey bees, at least in some environments and crops, is stingless bees. Stingless bees (Apidae; tribe Meliponini) are a group of over 500 species native to tropical and subtropical regions. This diverse group has a wide array of habitats and nest designs, many of which differ substantially from honey bees, as summarized by Michener [5]. However, all stingless bees share the eusocial lifestyle with honey bees, wherein hundreds or thousands of individuals live together in colonies with a single reproductive queen [5]. This is one of the key traits that make them useful as managed pollinators—large numbers of individuals can be contained within a single hive and transported to wherever their pollination services are needed [6]. Stingless bees are polylectic and are effective pollinators of a number of crops of tropical and sub-tropical origin, such as macadamia, mango and avocado, often exceeding the pollination efficiency of honey bees in these crops [7,8,9]. Some species of stingless bees can also pollinate crops such as tomato, which require ‘buzz pollination’ and for which honey bees are poor pollinators, and are becoming increasingly used in the greenhouse production of such crops (e.g., [10]).

Stingless beekeeping—meliponiculture—has a long and varied history in different parts of the world. In Australia and Africa, people native to these regions have extracted honey and other useful products from stingless bee colonies for millennia; however, there is no record of the widespread active management of colonies in these areas prior to the 20th Century [6,11]. In parts of India, Thailand and Southern China, stingless bee colonies have historically been collected from the wild and kept in specific locations to allow easier access to the resources they produce; however this practice was not historically widespread [12,13,14].

The most developed traditional meliponiculture practices originated in Central and Southern America, the center of stingless bee diversity. The management of *Melipona beecheii* in hives consisting of logs or ceramic vessels to produce honey and cerumen has been practiced by Indigenous populations in Meso-America for thousands of years [15]. More recently, they have been used in the pollination of some locally important crops such as avocado, rambutan and coffee [8]. However, unlike apiculture (the keeping of honey bees), meliponiculture has not spread globally as stingless bees are restricted to warmer climates.

This means that stingless bees are far less researched than honey bees, and this shortfall needs to be addressed if this group is to play an expanded role as managed pollinators in agricultural crops. One area where knowledge is needed is their pests and diseases, particularly under managed conditions. Currently, arthropod pests such as varroa mite, *Varroa destructor*, and microbial pathogens (including those vectored by these pests) are amongst the biggest limiting factors affecting apiculture [16], and an expanded stingless bee industry will also likely have to deal with similar issues. Thus, this paper seeks to elucidate, through the means of a comprehensive review of the published literature, what research has been undertaken into pests and diseases of stingless bees and where knowledge gaps exist.

## 2. Materials and Methods

This study comprises a comprehensive review of the published research literature on pests and diseases of wild and managed stingless bees. While a large number of popular stingless beekeeping guides exist (e.g., [6]), many of which describe pest and disease impacts and provide advice on their management, our review focused exclusively on assessing the completeness of the primary research literature related to these matters.

We assessed every published study that reported on stingless bee pest and disease issues that met our search criteria; however, due to the substantial differences in the data types, aims and methods of these studies, it was not possible to carry out a quantitative meta-analysis. Thus, this work represents a qualitative overview of the current state of the literature.

Searches for relevant studies were carried out in May 2025 using the Web of Science database, with 15 separate searches performed. All used the terms

“stingless bee” OR “stingless bees” OR melipon*

in addition to one of the search strings outlined in Table 1 within the ‘topic’ field. Searches 1 and 2 were carried out for the general topics of pests and diseases in stingless bees, with searches 3–15 examining the pest and disease groups highlighted by findings from the general searches as well as information contained within the popular literature. To gain an understanding of how the number of findings for stingless bees compared to those for honey bees, these searches were also repeated with the stingless bee-related terms noted above, replaced with

Apis OR honeybee* OR “honey bee” OR “honey bees”

All stingless bee searches were originally carried out in English, then repeated with Spanish and Portuguese translations of the same search terms (translated search terms shown in Table S1).

All results from the stingless bee searches were subjected to a title screening, and papers obviously unrelated to the topic(s) of interest were removed. Following this, abstracts for the remaining documents were assessed for relevance, with those that did not meet inclusion criteria (as outlined in Table 2) excluded and those remaining subjected to full-text reading. Results from the honey bee searches were not screened, but the numbers found were noted for comparison with the stingless bee findings.

For articles that were subjected to full-text reading, their reference lists were searched for other relevant articles.

## 3. Results and Discussion

A total of 75 studies were found that met inclusion criteria. The full list of included studies is shown in Table S2. Apart from Search 6, which focused on a pest genus believed to be a specialist in stingless bees (the parasitic wasp genus *Syntretus*) and Search 14, which returned very few results overall, all other searches resulted in substantially more results for honey bees than for stingless bees (Table S3).

### 3.1. Pests

**Figure 1:**
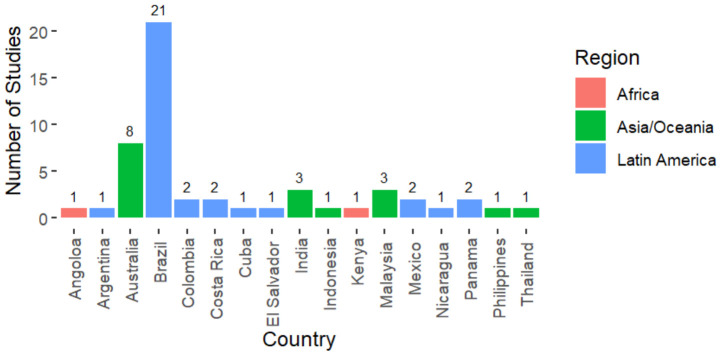
Pest studies found by country. Total number of studies exceeds 48 as some studies examined pests in multiple countries.

A total of 48 studies focusing on pests were found that met inclusion criteria. The countries of origin and the subject pest(s) of those studies are shown in Figure 1 and Table 3, respectively.

The only study that systematically catalogued pests and diseases of stingless bees was Dias de Freitas et al. [17], who utilized survey data from 344 stingless beekeepers throughout Brazil. In that survey, 75.1% of stingless beekeepers reported pests impacting their colonies, with the most common being phorid flies (Diptera: Phoridae), reported to affect 59.6% of stingless beekeepers over a two-year period. As expected based on the history of meliponiculture, the majority of studies (32/48) reported research fully or partly carried out in Latin America.

We found three studies examining the impact of parasitoid or specialist predatory wasps on stingless bees [18,19,20]. However, in each case, these studies examined wasps that attacked only individual stingless bees outside colonies, not attacking or reproducing within hives. Thus, these studies did not meet the inclusion criteria outlined in Table 2 and no studies that met our selection criteria focusing on wasps were found.

#### 3.1.1. Cleptoparasitic Bees

The most widely reported pests of stingless bees were other stingless bee species, with 10 studies examining the impacts on stingless bee hives of attacks from this group. These studies covered two types of attack: resource robbing and nest usurpation, actions collectively referred to as cleptoparasitism or cleptobiosis [21]. In resource robbing, the attacking bees remove provisions and other materials from within a host hive and take them for use in their own hive. In nest usurpation, all adult bees from the host colony are killed or expelled from their hive, after which the attacking colony reproduces by sending its own workers and a newly mated queen into the colony, making use of all provisions, the nesting site and, in some cases, emerging workers from the host colony [21].

Cleptoparasitism can be either a facultative foraging strategy, when carried out by bees also able to forage on floral resources, or an obligate strategy. Obligate cleptoparasitism is seen in all described species of the stingless bee genera *Lestrimelitta* and *Clepotrigona*; these bees have lost the corbiculae that other bees use to gather pollen and are believed to never visit flowers, relying entirely on nest robbing to meet the nutritional needs of their colonies [22].

Michener [23] reported on 10 separate attacks by *Lestrimelitta limao* on *Trigona testaceicornis perilampoi* in Panama. Individuals from the host species were reported to avoid the hive, while *L. limao* was present. After the raids, host activity was observed to rapidly return to normal, though this study did not report on the condition inside the hive following this attack, nor whether the colonies collapsed or recovered. Sakagami et al. [22] reported on observations of 90 raids by *L. limao* on 17 species of stingless bees in Panama and Brazil; these raids typically saw all brood provisions removed from host hives (resulting in the death of developing brood), although honey and pollen pots were usually only partially depleted and host hives collapsed in only 6 of the 90 cases examined.

Mascena et al. [24] reported on two attacks by *Lestrimelitta rufa* on *Melipona quinquefasciata* colonies housed in artificial hives. While the hosts were observed to narrow the nest entrance and place additional guard bees at this point, these responses seemed to be ineffective, with the raid resulting in most honey and pollen pots being removed, many dead workers and, in one case, a dead queen. One hive collapsed while the other survived after human intervention, which included the removal of the attacking bees. The authors suggested that the lack of an effective host response may be due to this species relying on its naturally cryptic nests to avoid raids, an advantage that is lost when the species is kept in an artificial hive.

Costa et al. [25] reported on resource-stealing raids by the facultatively cleptoparasitic stingless bee *Melipona fuliginosa*, observing attacks on both *Melipona paraensis* and *Melipona fasciculata* in a multi-species meliponary in Brazil. In the case of *M. paraensis*, the single attacked colony appeared to offer no resistance, with all foraging, guarding and egg-laying in the colony ceasing for the five days that the researchers observed the raid. Honey and pollen resources were stolen from the colony, but, after the attack ended, *M. paraensis* behavior returned to normal and the colony recovered naturally. In the case of *M. fasciculata*, some evidence of resistance was seen in the five attacked hives. Workers of both *M. fasciculata* and *M. fuliginosa* were seen grappling, and many dead workers were found outside the hives, with the number of dead host workers exceeding those of attackers by an order of magnitude. After the attack, the attacked hives were missing all honey pots and all flying workers. The hives ultimately survived, but only after human intervention, in the form of supplemental feeding and the trapping of secondary pests, was carried out.

Rech et al. [26] observed stingless bees of the species *Duckeola ghilianii* and *Melipona fulva* attacking *Lestrimelitta rufipes* workers, robbing a hive of *Scaptotrigona* sp. (a genus considered to be particularly vulnerable to resource robbing attacks). In contrast to other species, *D. ghilianii* were able to kill *L. rufipes* workers without suffering any observed mortality themselves. The authors noted that this was unlikely to be a case of interspecies altruism, but rather a very sensitive response by the species involved to the odors produced by attacking *L. rufipes.* Although this work was simply an incidental case study, and did not report on the level of damage ultimately sustained by the *Scaptotrigona* sp. hive, the authors suggested that placing hives of vulnerable species near highly defensive species may be a method to reduce the impacts of resource robbing.

Portugal-Araujo [27] reported on experimentally placing a colony of the obligate cleptoparasite *Cleptotrigona cubiceps*, found naturally occurring in a tree hollow and relocated to an artificial hive, within 50 m of three hives of *Trigona braunsi* in a meliponary in Angola. The study reported that *C. cubiceps* robbed both food (honey and brood provisions) and building materials (propolis and cerumen) from the host hives, killing many workers in the process, but, after raids were concluded, hives still contained some workers and provisions. Whether these hives collapsed or recovered was not reported.

In addition to the loss of resources and mortality of individuals caused by resource robbing, Segers et al. [28] identified an indirect evolutionary impact of the presence of cleptoparasites. The stingless bee *Tetragonisca angustula*, native to Central and South America, has a morphologically distinct soldier caste, larger than typical workers, which guards nests from intruders. It is believed that the threat posed by cleptoparasites is one of the major selective pressures responsible for the evolution of caste differentiation in this species [29]. Segers et al. [28] observed that, in regions of Brazil where cleptoparasitic bees are more common, *T. angustula* colonies had a higher ratio of soldiers to workers amongst their adult population than in regions where they are rare, while colonies artificially exposed to chemical cues from *L. limao* deployed greater numbers of soldiers to guard their hives for several months after the exposure. As soldiers are larger than workers and do not forage, this represents a cost to colony fitness due to the presence of robber bees.

Nest usurpation was reported in three studies, all focusing on Australian stingless bees from the *Tetragonula* genus. Cunningham et al. [30] observed the usurpation of an artificial hive occupied by *Tetragonula carbonaria* by *Tetragonula hockingsi.* The process occurred over four months, during which time numerous fighting swarms were observed between the two species, resulting in thousands of worker deaths on both sides, before the hive ultimately became occupied by a healthy colony of *T. hockingsi.* The same work also reported on a five-year study in which an average of 260 *T. carbonaria* and 21 *T. hockingsi* managed colonies were observed annually, finding 3% of *T. carbonaria* and 5% of *T. hockingsi* hives to be usurped by the opposite species over that period.

Xia et al. [31] carried out a similar observation, tracking the species identity of 48 *T. carbonaria* colonies and 11 *T. hockingsi* colonies naturally occurring in an area of bushland over five years. Over the course of this period, eight *T. carbonaria* colonies were replaced by *T. hockingsi* colonies, while the reverse was never observed. The authors noted that these changes in species composition are unlikely to be cases of a new species moving into an abandoned nest, as such nests are typically left vacant due to the risk of fungal diseases in empty hives, and thus these findings are evidence of aggressive usurpation.

Lau et al. [32] reported on multiple swarm fights with thousands of casualties preceding the usurpation of a *T. carbonaria* colony over a period of 63 days. In this case, fights were observed with conspecific colonies, as well as with *T. hockingsi.* The hive was eventually occupied by *T. hockingsi* but this usurping colony collapsed within two weeks, leaving no living bees of any species present within the hive. Throughout the period that the hive was observed, no evidence of the removal of provisions by attacking bees was recorded, illustrating that usurpation is a substantially different activity from the resource robbing discussed above.

#### 3.1.2. Beetles

Small Hive Beetle

The small hive beetle (SHB—*Aethina tumida*, Coleoptera: Nitidulidae) is native to sub-Saharan Africa but in recent decades has spread across most of the world [33]. In its native range, it reproduces within hives of African sub-species of honey bees and is primarily a scavenger with little negative impact. However, outside its native range, it is a major pest of managed and wild honey bees; larvae feed on hive food supplies and brood, and their waste accumulates and ferments within hives [33].

Stingless bees can be impacted by this pest but appear to be less appealing hosts than honey bees. Neumann et al. [34] found SHBs entered honey bee hives in preference to hives of *T. carbonaria* in a ratio of 13:1, while Bobadoye et al. [35] found odors from honey bees were more attractive to SHBs than those from two species of Kenyan stingless bees, *Meliponula bocandei* and *Meliponula ferruginea*.

Stingless bee defensive behaviors also seem to be effective against SHBs. Studies by Greco et al. [36] and Halcroft et al. [37] on the Australian species *T. carbonaria* and *Austroplebeia australis*, respectively, found both were able to resist SHBs through means such as ‘mummifying’ adults (encasing them in resin), consuming eggs and ejecting larvae from nests. These actions may be more effective than the stinging behavior employed by honey bees, as SHBs’ strong cuticles and the adoption of defensive postures render them resistant to stings. Also, within honey bee hives, SHBs are able to hide in cracks out of the reach of the bees, a behavior that is less effective against the smaller stingless bees [37].

SHBs appear to become a serious threat to stingless bees primarily when hives are weakened by other factors. Nacko et al. [38] found that, after exposure to heatwave conditions (four days of temperatures over 40 °C), two *T. carbonaria* hives became infested with SHBs. Seven other hives kept in the same area, but that were more shaded, did not become infested. Similarly, Cervancia et al. [39], Loriga Pena et al. [40] and Pereira et al. [41] reported on the first known cases of SHBs within stingless bee hives in the Philippines, Cuba and Brazil, respectively, and in each case only found these beetles in hives that were already weakened prior to invasion. Toledo-Hernandez et al. [42] reported on the first finding of SHBs in stingless bee hives in Mexico—that work did not report any specific stressors, as the affected hive had not been subject to prior systematic monitoring.

The above studies suggest that SHBs are typically a relatively minor pest of stingless bees. The relatively high level of reporting on SHBs may thus be indicative of their novelty as a recently introduced species in the various regions where the studies were conducted.

Other beetle species

We found six studies that reported on beetles other than SHBs invading stingless bee hives. Krishnan et al. [43] reported the presence of the beetle *Haptoncus luteolus* (Nitidulidae) in a single hive of each of three Malaysian stingless bee species: *Trigona thoracica*, *Heterotrigona itama* and *Tetragonula laeviceps*. Damage to *H. itama* and *T. thoracica* hives was noted as being minor, in contrast with *T. laeviceps*, where hundreds of beetle larvae, pupae and adults were found in brood, pollen and honey pots, and the colony ultimately collapsed.

Camenzind et al. [44] described the parasitic activity of the beetle *Procoryphaeus violaceus* (Histeridae), after finding it to be present in collapsed colonies of *Teteragonula pagdeni* in Thailand. *P. violaceus* produced impacts similar to those observed in SHB infestations, with larvae feeding on colony food supplies, destroying wax, pollen and honey pots, and excreting throughout the hive. Inoue et al. [45] reported a related species, *Procoryphaeus wallacei*, associated with the loss of 12 established colonies of *Tetragonula minangkabau* over a five-year period in fruit tree plantations in Indonesia.

Carvalho et al. [46], Meneses et al. [47], and Depintor and De Jong [48] all reported on the invasion of stingless bee hives by clown beetles—*Hololepta reichii* (Histeridae)—in Brazil. Meneses et al. [47] recorded this beetle in 41 colonies across five bee species in a multi-species meliponary, 24 of which ultimately collapsed. Depintor and De Jong [48] reported the loss of a colony of *Tetragonula clavipes* following invasion by both *H. reichii* and black soldier flies (*Hermetia illucens*) in another multi-species meliponary in Brazil. Carvalho et al. [46] found a small number of *H. reichii* adults in a hive of *Scaptotrigona xanthotricha* that had recently been relocated from a natural situation to artificial housing in Brazil. While they had caused no apparent damage within the hive, under laboratory conditions, the adult beetles readily preyed upon *S. xanthotricha* brood and provisions, indicating a causal link was possible between this beetle and the colony collapses reported by Meneses et al. [47] and Depintor and De Jong [48].

These studies mostly do not report the relationship between external stressors and beetle invasion as was typically described in the SHB studies, likely due to the different nature of these studies. Apart from Inoue et al. [45], all are small case studies, reporting on the impacts of beetle invasions after they had occurred, making it difficult to draw inferences about what events may have preceded the reported invasion. Inoue et al. [45] represents a long-term study in which hives were systematically monitored but does not report on other potential stressors.

Coleoptera is the most diverse arthropod order, and beetle pests of social bees occur in at least four other families, in addition to Nitidulidae and Histeridae mentioned above (Meloidae, Ripiphoridae, Cetoniidae and Cleridae: [49,50,51]). Given the opportunistic nature of these studies, and the fact that three of the six studies on beetles other than SHB represent the only publications regarding the impact of their focal species on stingless bees [43,44,45], it seems likely that a large knowledge gap exists and other beetle species may be pests of stingless bees, but have not yet come to the attention of researchers.

#### 3.1.3. Mites

Mites (Acari) can be major pests in honey bees. Of particular note is the cosmopolitan, parasitic *Varroa destructor*, considered to be the single largest cause of honey bee colony loss worldwide, with harm caused due to both its feeding on bee hemolymph and fat bodies and its ability to vector diseases [52,53]. While *Varroa* mites do not attack stingless bees, several other mite species are known or suspected to impact this group.

Sanchez-Quilindo et al. [54] systematically collected over 2000 individuals from wild and managed colonies of honey bees and three stingless bee species (*Partamona peckolti*, *Paratrigona eutaeniata* and *Tetragonisca angustula*) in the Cauca and Valle del Cauca departments, Colombia, to assess the presence of parasitic mites. They found the hemolymph-feeding ectoparasitic mite *Leptus alberti* (Erythraeidae) attached to 5% and 23% of honey bees in the two departments respectively, and on 0.3% and 4.2% of stingless bees. Santos Louzado das Neves et al. [55] also surveyed for the presence of *Leptus* mites, carrying out systematic examinations of seven managed hives of *Melipona scutellaris* in Brazil. In six of the hives, small numbers of individual mites (<30) were found to be attached to adult bees at various times throughout the year. Martínez et al. [56] systematically searched for mites attached to stingless bees in specimen collections gathered over several years in Misiones province, Argentina. They found 16 mites (all *Leptus* spp.) on 4 species of bees from a sample of 1829 individual bee specimens from 12 species. None of these studies examined the health consequences to individual bees or colonies of parasitism by these mites; however, ectoparasitic *Leptus* spp. are known to be vectors of bacterial diseases in honey bees [57].

Like many of the studies found on beetles, the remaining six studies on mite interactions with stingless bees were all opportunistic case studies, reporting on isolated observations rather than being part of a systematic research project.

Bassini-Silva et al. [58] reported on the finding of numerous *Leptus adaminae* parasitising a single *Melipona quadrifasciata* individual from a managed hive in Brazil. As above, no analysis of the survival or health impacts was conducted.

Macias-Macias and Otero-Colina [59] reported finding an infestation of the mite *Pyemotes tritici* (Pyemotidae) in a collapsed colony of *Melipona colimana* in Mexico. This followed a very rapid decline in colony health; the hive appeared healthy just two days before it collapsed. However, the authors suggested that the mites had likely been present, but unnoticed, for some time prior.

Menezes et al. [60] also reported on infestations by *P. tritici* and implicated them in the collapse of five stingless bee colonies (four *T. angustula* and one *Frieseomelitta varia*) in a multi-species meliponary in Brazil. Collapsed colonies were found with food stores intact but many mites attached to dead adult bees and larvae. These mites appeared to have been spread by the transfer of hive components by beekeepers; despite around 40 other hives being in close proximity, only these 5 hives collapsed, suggesting that this mite has relatively poor dispersal ability.

da Silva et al. [61] reported on the collapse of six *F. varia* colonies from a Brazilian meliponary that were found to contain the mite *Tyrophagus putrescentiae* (Acaraidae). The mites were observed feeding on live and dead brood, both in the hives and in an artificial brood rearing medium maintained under laboratory conditions. While *T. putrescentiae* is typically considered to be mycophagous, it has also been previously reported to prey on insects [62,63]. The authors suggested that this mite, along with cold, dry weather, was responsible for the collapse of the colonies and that, as in Menezes et al. [60], the infestation was introduced by poor hygiene practices on the part of researchers managing the hives.

The only studies found detailing negative impacts of mites on stingless bees from outside Latin America were two studies from India by Vijayakumar et al. [64] and Devanesan et al. [65], both of which were case studies of mites on *Tetragonula iridipennis*. Vijayakumar et al. [64] outlined the effects of the infestation of a hive by *Carpoglyphus lactis* (Carpoglyphidae), a mite that is typically considered a pest of stored food products. The authors found the mites present in managed colonies and subsequently reared them under laboratory conditions. The mites appeared to consume larval food supplies, damaging brood cells and pupae in the process. This led to the death of bees in these life stages through starvation and/or desiccation, and the colony collapsed within a month. Devanesan et al. [65] reported on the observation of *Amblyseius* sp. (Phytoseiidae) mites feeding on hive pollen stores, with the bees subsequently abandoning the colony.

As with beetles, reports on mite infestations of stingless bees are sparse. The nine papers examined here cover at least five species of mites, with five of the papers serving as simple case studies, reporting the impact of mites on stingless bees after infestations fortuitously came to the attention of researchers. Mites are known to play a very significant role as pests of honey bees, and it is unclear if the much smaller research base regarding their effects on stingless bees is due to their lesser impacts or a genuine knowledge gap.

#### 3.1.4. Flies

Phorid Flies

Phorid flies (Diptera: Phoridae) are a family containing over 2500 species, many of which are parasites of a range of invertebrates [66], including both social and solitary bees [67,68]. The relationship between phorid flies and stingless bees is the oldest known example of a parasite–host relationship amongst insects, with a pair of these organisms found preserved together in 80-million-year-old amber [69]. Importantly, phorids were identified by Dias de Freitas et al. [17] as the single most common pest of managed stingless bees in Brazil. Various species of phorids have been observed feeding on both hive food provisions and bees of all life stages as both parasitoids and in-hive predators [70]. Despite their importance, we found just eight studies reporting on the relationship between phorid flies and stingless bees that met our search criteria.

An early description of this relationship can be found in Salt [71] from 1929. This work reports the phorid species *Pseudohypocera nigrofascipes* in hives of stingless bees in both Brazil and Colombia. What impact these flies had on the hives is unclear but the fact that phorid pests of stingless bees have been the subject of scientific study for almost a century is indicative of their importance.

Brown [72] reported on observations of females of the phorid species *Apocephalus apivorus* attempting to oviposit in drones of the stingless bee species *Cephalotrigona capitata* gathered as part of a drone swarm outside a hive in Costa Rica. *C. capitata* drones and workers were collected and, while almost half of all drones were found to have been parasitised by *A. apivorus*, none of the workers were (although 1 of the 100 workers collected was found to be parasitized by a different phorid—*Melaloncha* sp.).

Simoes et al. [73] observed attacks by the phorid *Melaloncha sinistra* on the stingless bee *Scaptotrigona postica*, noting that adult females would attempt to oviposit in foraging workers at hive entrances but not venture into hives themselves. Simoes et al. [73] also collected foragers of both *S. postica* and several other co-located stingless bee species and found that, while an average of 10% of *S. postica* workers were parasitized by *M. sinistra*, no other species were, leading the authors to suggest this phorid may be a specialist on *S. postica*. In some colonies, up to 37% of *S. postica* foragers were found to be parasitized; however, in no cases did a colony appear to collapse because of this parasitism.

Disney and Bartareau [74] provided a description of the ecology of the phorid species *Dorniphora trigonae*. Both larvae and adults of this species were found in hives of *T. carbonaria* in Australia, and the presence of pollen grains within their guts confirmed that they had consumed hive food supplies. Gut content analysis in the same study of another phorid, *Pseudohypocera kerteszi*, from stingless bee hives in Brazil and Nicaragua found pollen grains within the guts of this species too, confirming that they shared similar feeding habits.

Correia et al. [75] also investigated *P. kerteszi*, examining its ability to enter hives of *Scaptotrigona tubiba* in Brazil. They found that *S. tubiba* guards typically failed to prevent the entry of adult *P. kerteszi* into their hives, despite being able to repel heterospecific stingless bees highly effectively. The authors investigated whether this may be due to cuticular hydrocarbons serving to deceive guards but found no evidence of this, instead suggesting that the phorids were able to evade guard bees using their speed and small size.

Robroek et al. [76] observed the progress of the invasion of a hive of *M. beecheii* in El Salvador by *P. kerteszi*. Adult phorid females were observed accessing the hive through the entrance and initially oviposited in pollen stores and the hive’s waste dump, where hatching larvae consumed both pollen and waste. As these supplies were consumed, eggs were increasingly laid in the brood combs, where hatching phorid larvae fed on bee brood. In the final stage of the invasion, after brood combs had disintegrated, phorid larvae were observed in the honey pots.

Moretto [77] and Martins de Oliveira et al. [78] also focused on *P. kerteszi*, both investigating the efficacy of phorid traps commonly used by beekeepers in Brazil. Both found vinegar-based traps highly effective at catching phorids, whilst not being attractive to bees.

Despite both popular sources (e.g., [6]) and Dias de Freitas et al. [17] confirming their status as highly destructive pests, the eight studies outlined above are the only primary reports we are aware of examining the relationship between phorids and stingless bees. This highlights that, as with the other pests previously discussed, there is a substantial knowledge gap regarding this family and further research is needed to assist stingless beekeepers in managing this pest.

Soldier Flies

Black soldier flies (*Hermetia* spp., Diptera: Stratiomyidae) typically feed on decomposing organic matter and have been the subject of significant research on their ability to process waste biomass [79]. While they are known to feed on detritus deposited outside stingless bee hives [80], their status as pests of stingless bees is uncertain, with differing conclusions reached amongst the four studies examined.

Hashim et al. [81] reported large numbers of *Hermetia illucens* in 250 collapsed hives of the stingless bees *Geniotrigona thoracica* and *H. itama* in Malaysia. While Hashim et al. [81] implicated the soldier flies for the collapse, Ivorra et al. [82] disputed this finding, suggesting that the hives had already become ‘spoiled’ and that soldier flies had simply been attracted to the smell of decomposition. Ivorra et al. [82] also disputed Hashim et al. [81]’s identification of the soldier fly species involved, providing evidence that it was in fact *Hermetia fenestrata*. Ivorra et al. [82] reported finding both *H. illucens* and *H. fenestrata* larvae in a nest of *G. thoracica* from Malaysia that had already begun to decompose. The larvae were raised to adulthood on decomposing nest material, without any evidence that they consumed bee brood or pupae, leading Ivorra et al. [82] to suggest that the soldier fly species involved are not pests of stingless bee, but simply opportunistic scavengers of decaying hive contents.

Depintor and De Jong [48] also reported finding large numbers of *H. illucens* larvae in a collapsed colony of *T. clavipes* in Brazil, which was also infested by clown beetles (discussed above). Contrary to the findings of Ivorra et al. [82], Depintor and De Jong [48] observed *H. illucens* feeding on bee brood; however, they also suggested that the hive was likely weakened by beetles prior to the arrival of *H. illucens*. Devanesan et al. [65] also reported finding *H. illucens* larvae in a hive of *T. iridipennis* in India, finding the colony to have collapsed, which the authors attributed to the soldier flies.

The above studies leave the role of soldier flies as pests of stingless bees uncertain. On balance, they seem unlikely to act as hive invaders on their own, but their role in contributing to the collapse of already weakened hives remains unclear.

#### 3.1.5. Other Pests

In addition to the above pests, we found two studies addressing the impacts of wax moths and a single study examining mantisfly impacts on stingless bees.

There are two species of wax moth (Lepidoptera: Pyralidae), the Greater wax moth (*Galleria mellonella*) and the Lesser wax moth (*Achroia grisella*), that are widespread pests of honey bees, particularly in tropical and sub-tropical regions, with the former species the more important [83]. However, we are only aware of two studies that report the pest status of wax moths in relation to stingless bees.

While noting that they were aware of numerous reports of unidentified moths being found in Brazilian stingless bee hives, Cepeda-Aponte et al. [84] reported on the first conclusively identified wax moth larvae, *A. grisella*, in stingless bee hives. The hives of *Melipona bicolor bicolor* and *M. quadrifasciata* in which these larvae were found were weak, and it was found that *A. grisella* larvae could be raised to adulthood on a diet of hive detritus. This led Cepeda-Aponte et al. [84] to conclude that, like several of the other possible pests discussed in this review, the presence of this species was more likely a symptom of an unhealthy hive, rather than the cause. Gopinatha and Basavarajappa [85] reported finding *G. mellonella* in 2.1% of surveyed stingless bee hives in the Indian state of Karnataka. They were observed to lay their eggs in hives, with the larvae consuming developing brood; however, the relationship between infestation with this pest and hive collapse was not reported.

Maia-Silva et al. [86] provide the only study of the parasitism of stingless bee hives by mantisflies (Neuroptera; Mantispidae). The work reports on *Plega hagenella* (subfamily Symphrasinae) infesting Brazilian *Melipona subnitida* hives, with *P. hagenella* larvae found occupying brood cells and feeding on bee larvae. Maia-Silva et al. [86] carried out experiments assessing behavioral interactions between *M. subnitida* and *P. hagenella*, observing that post-eclosure adult mantisflies introduced to hives were immediately attacked and killed by bees, but pharate adults were removed by workers unharmed, in a similar manner to the way in which waste is removed from hives. Maia-Silva et al. [86] concluded that this was evidence that *P. hagenella* had evolved chemical cues deceptive towards *M. subnitida*.

Beyond the work of Maia-Silva et al. [86], reports of mantisflies parasitizing bee hosts in general are rare. A review by Snyman et al. [87] suggests that the subfamily Symphrasinae may be a specialist parasite of social bees, but notes that knowledge is limited. Thus, while Maia-Silva et al. [86]’s work shows that mantisflies can impact stingless bee colonies when circumstances lead to their invasion, it is unclear how widespread harmful mantisflies are and, thus, how severe their impacts may be on stingless bee populations overall.

### 3.2. Diseases

Viral, bacterial and fungal diseases are major causes of colony loss in honey bees and have been widely studied for more than 50 years [88,89]. Several dozen diseases are known to occur in honey bees [88,89], but it has been suggested stingless bees may be less susceptible to microbial disease due to a number of factors.

Studies in various stingless bee species have shown they exhibit superior hygienic behavior to honey bees, being able to locate and remove dead brood from hives at a quicker rate than typical honey bee colonies [90,91]. It has also been suggested that symbiotic bacteria and anti-microbial properties of stingless bee honey and propolis can help colonies resist diseases [92,93]. Additionally, while honey bees reuse brood cells, with queens laying new eggs in old cells after larvae vacate, stingless bees use such cells only once, dismantling them after use [91], and this may also help to prevent diseases. However, the only study we are aware of that compares disease incidence in honey bees and stingless bees, the survey of Brazilian beekeepers carried out by Dias de Freitas et al. [17], reported symptoms that may be indicative of diseases at similar rates in the two groups of bees.

**Figure 2:**
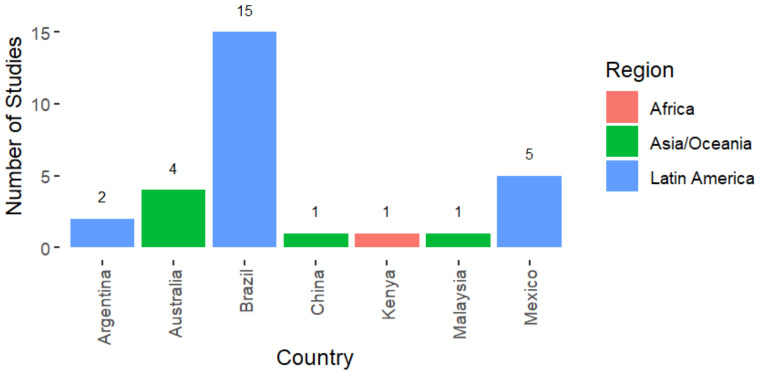
Disease studies found by country. Total number of studies exceeds 28 as some studies examined diseases in multiple countries.

**Figure 3:**
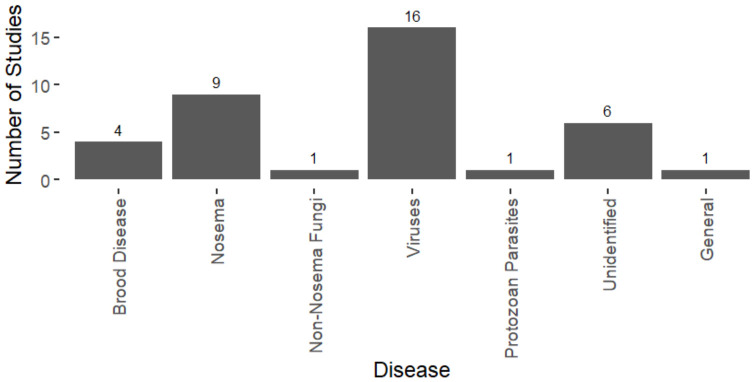
Disease studies found by disease type. Total exceeds 28 as some studies examined multiple types of disease. “General” refers to the work of Dias de Frietas [17], who carried out a survey of Brazilian stingless bee keepers on pests and diseases encountered.

A total of 28 studies that reported on diseases in stingless bees were examined, with the country of origin and disease types studied shown in Figure 2 and Figure 3.

#### 3.2.1. Brood Diseases

Honey bee colonies are impacted by a number of brood diseases such as European Foulbrood, American Foulbrood, Chalkbrood, Stonebrood and Sacbrood [94,95]. These diseases can be caused by bacterial, fungal or viral pathogens, but all result in brood death and can lead to colony collapse. We found four studies investigating brood diseases in stingless bees, all focusing on bacterial pathogens.

Shanks et al. [96] reported the first confirmed discovery of a stingless bee brood disease, finding a disease that caused larval death and reduced worker activity in the Australian stingless bee species *T. carbonaria*. The researchers isolated the causative bacteria, *Lysinibacillus sphaericus*, and reinfected an otherwise healthy hive, reproducing the symptoms and thus confirming Koch’s postulates. This disease is now commonly referred to as ‘Shanks Brood Disease’ (SBD).

Further research by Roy [97] into SBD found that it appears to be widespread across much of eastern Australia and also infects the species *T. hockingsi* and *A. australis*. However, SBD infections appeared quite rare amongst the general population of managed stingless bees in Australia and *L. sphaericus* was never found in healthy hives or adult bees. Roy [97] suggested the disease is more likely to be picked up from the environment than transmitted between hives, as *L. sphaericus* can be found in soil without being associated with bees.

Teixeira et al. [98] carried out a molecular analysis of a brood disease found in two multi-species meliponaries in Brazil that were observed to have discolored and dead brood, with some collapsed colonies. They identified *Melissococcus plutonius*, the bacterium responsible for the disease European Foulbrood in honey bees, to be present in five of the six species examined. The authors suggested the pathogen was likely transmitted via honey bee-derived products used for supplemental feeding.

The final study on brood disease is by Amirthalingam et al. [99], who systematically searched for a range of diseases in colonies of *H. itama* and *G. thoracica* from throughout Malaysia. This search included molecular tests for the presence of both SBD and European Foulbrood but no evidence for the presence of either pathogen was found.

#### 3.2.2. Nosemosis and Other Fungal Diseases

Nosemosis is a disease caused by *Nosema*, a genus of pathogenic fungus of class Microsporidia (a group previously classified as protists [100]). *Nosema* infects bees and reproduces within the gut, causing dysentery, gut abrasion, immune suppression and lethargy in individual bees, potentially leading to colony collapse in social species [100]. It is spread via the fecal–oral route and can be transmitted between colonies of the same or different species through the contamination of floral resources. It is known to infect honey bees, bumble bees and solitary bees and has a global distribution [100]. However, how widespread Nosemosis is in stingless bees, and what impacts it may have on them, is less well known; we found nine studies investigating this.

Both Nunes-Silva et al. [101] and Cristina Dias et al. [102] sampled stingless bees across southern Brazil (six species examined by Nunes-Silva et al. [101], and a single species, *F. varia*, was examined by Cristina Dias et al. [102], who used molecular techniques to test for pathogens, including *Nosema*. In both cases, no evidence of *Nosema* was found in any stingless bees, despite it often being found in co-located honey bees. Nunes-Silva et al. [101] suggested the anti-microbial properties of stingless bee propolis may have been responsible for these negative results. Similarly, Nkoba et al. [103] sampled stingless bees from 10 species across Kenya, while Brettell et al. [104] sampled a single species (*T. carbonaria*) in Australia and in both cases also found no evidence of *Nosema*. This contrasts with the work of Porrini et al. [105] and Roy [97], who tested for *Nosema* in pooled samples of stingless bees from across Brazil and Argentina [105] and Australia [97], finding this fungus to be present in 93% and 10% of samples, respectively. However, neither study determined if this pathogen was reproducing within the bees and/or causing any symptomatic effects, or merely present.

Teixeira et al. [98] tested for *Nosema* in multiple stingless bee species in Brazilian meliponaries. They found *Nosema ceranae* in the brood of one species, *Melipona marginata*, as well as in honey bee-derived products used as supplemental food for the colonies, which the authors suggested was the likely source of infection. Whether *Nosema* was reproducing or causing symptomatic effects in these bees was not examined.

Guimaraes-Cestaro et al. [106] used both molecular and microscopic techniques to test for *Nosema* in three species of stingless bee (*Nannotrigona testaceicornis*, *T. angustula* and *Tetragona elongata*) from hives in Brazil, with foraging ranges overlapping honey bees known to be infected by *N. ceranae*. When testing whole worker bees, they found *N. ceranae* to be present in around 50% of individuals of each species. However, when examining the midguts of these bees (the system that must be infected for this disease to become symptomatic), they found no evidence of *Nosema*. The authors suggest this meant the stingless bees examined were carrying spores externally without becoming infected, and suggested this was also likely the case in the studies carried out by Nunes-Silva et al. [101] and Porrini et al. [105], with the latter study having surface sterilized bees prior to analysis.

We are only aware of two studies that directly examined Nosemosis symptoms in stingless bees. One is Macias-Macias et al. [107], who fed workers of the Mexican stingless bee species *M. colimana* sugar syrup containing *N. ceranae* spores, either alone or in combination with a sub-lethal dose of insecticide. At 14 days after treatment, they found that, in the group fed *N. ceranae* spores without insecticide, the spore count per bee exceeded the initial inoculation, providing evidence that *N. ceranae* reproduced within those bees. These bees also showed signs of immunosuppression; however, longevity was only significantly reduced in the combined *Nosema* and insecticide treatment, with bees that received either *N. ceranae* spores or insecticide alone having survivorship rates similar to untreated controls. The authors concluded that this was evidence of these two stressors being most harmful in combination.

The other study is by Purkiss and Lach [108], who fed *N. ceranae* spores to adults of the Australian species *T. hockingsi*. They found *N. ceranae* reproduced within the bees and that bee longevity was significantly reduced compared to the control group. They also found *N. ceranae* spores present in the guts of *T. hockingsi* that had fed on flowers where *N. ceranae* was present due to foraging by infected honey bees. However, *N. ceranae* appeared to have somewhat limited transmissibility as not all bees exposed to spores in their food became infected. The authors suggested this laboratory-based study was the ‘best case’ scenario for infection and that, in more realistic scenarios, infection rates may be even further reduced.

These studies highlight that our understanding of how readily *Nosema* can infect stingless bees under natural conditions is limited. Additionally, hive-level impacts have not been examined. Unlike honey bees, where adults defecate outside of hives, stingless bees defecate in a designated waste area within their hive, with eventual management of this waste varying between species [109]. It is possible that, in contrast to other hygienic behaviors exhibited by stingless bees, this behavior may make this group more vulnerable to fecal-transmitted diseases than honey bees.

The only study found that examined fungal pathogens other than *Nosema* in stingless bees is by Amirthalingam et al. [99]. This study was carried out after the authors isolated a pathogen they believed was responsible for the collapse of a hive of *H. itama*. The agent was identified as *Aspergillus caelatus*, and it was experimentally found to be capable of causing complete brood mortality in both *H. itama* and another Malaysian stingless bee species, *G. thoracica*.

#### 3.2.3. Viruses

Viruses were the most widely researched category of stingless bee pathogens, with 16 studies found. All examined one or more of a group of seven viruses commonly found in honey bees, namely, Deformed Wing Virus (DWV), Black Queen Cell Virus (BQCV), Acute Bee Paralysis Virus (ABPV), Chronic Bee Paralysis Virus (CPBV), Sacbrood Virus (SBV), Israeli Bee Paralysis Virus (IBPV) and Kashmir Bee Virus (KBV). Most of the studies used molecular techniques to test for the presence or absence of these viruses within stingless bees, as summarized in Table 4.

Table 4 illustrates that all the major viruses of honey bees have been detected in one or more species of stingless bee, with most studies suggesting this is a result of viral spillover. However, there remains an extensive literature gap regarding the impact of these viruses, as most studies did not assess whether virus presence was associated with symptoms in stingless bee hosts.

Only two studies systematically investigated virus symptoms in stingless bees. Fleites-Ayil et al. [112] fed particles of BQCV and two strains of DWV to adult *M. beecheii* workers. They found viral load increased in treated bees, indicating successful reproduction, and also found bee survival was significantly reduced in all three virus treatments. While DWV can result in symptoms such as shrunken wings, most adult honey bees infected with it appeared healthy but had reduced lifespans [119], so the findings of Fleites-Ayil et al. [112] are consistent with its most common impact on honey bees. In contrast, BQCV does not typically cause observable symptoms in adult honey bees [119].

Ueira-Vieira et al. [117] tested for the seven viruses listed in Table 4 in 10 hives of *M. scutellaris* within which beekeepers had reported increased bee mortality. All 10 hives tested positive to ABPV and collapsed within six months. Five other apparently healthy hives were also tested and returned negative results for all seven viruses. The authors stressed that their findings did not necessarily imply a direct causal relationship between the virus and the collapse and that the presence of the virus could be a result of the hives being weakened by other factors.

Two other studies detected viruses replicating within stingless bees without examining symptoms. Morfin et al. [114] found evidence of the replication of DWV and BQCV in *M. colimana* through observing negative strand RNA, while Tapia-Gonzalez et al. [116] also found evidence of the replication of DWV and BQCV in *M. colimana* and DWV in *Trigona fulviventrisa* and *Nannotrigona perilapoides*.

Additionally, Alvarez et al. [110], Cristina Dias et al. [102], Guimaraes-Cestaro et al. [106] and Roy [97] all noted that the bees examined in their research were not displaying obvious virus symptoms at the time of their experiments. However, in each case, this was just a general observation. Given that the symptoms of the viruses examined can be somewhat cryptic in honey bees [120], these observations do not provide conclusive evidence as to whether the relevant viruses can be symptomatic in stingless bees.

Three of the studies noted in Table 4 also examined other viruses beyond the seven listed. Fleites-Ayil et al. [112] also tested for the presence of Slow Bee Paralysis Virus, while Zhang et al. [118] tested for *Apis mellifera* filamentous virus and Brettell et al. [104] tested for Lake Sinai virus, with all three finding negative results.

Thus, while viruses are the most widely researched group of pathogens within stingless bees, and it is apparent that most common honey bee viruses can be detected in stingless bees, there is a major knowledge gap regarding whether or not they are harmful.

#### 3.2.4. Protozoan Parasites

We are aware of just one study that examined parasitic protozoans in stingless bees, the work of Nunes-Silva et al. [101], who tested for pathogens in the order Neogregarinorida and in the suborder Leishmaniinae (order Trypanosomatida). They found evidence of the neogregarine parasite *Apicystis bombi* in two stingless bee species (*Plebeia emerina* and *Tetragonisca fiebrigi*), despite its absence in local honey bees, and suggested it may have been present because of spillover from wild bumble bees. They did not assess whether *A. bombi* impacted the health of stingless bees. No evidence of Leishmaniinae was found within any of the six stingless bee species examined, despite the species *Lotmaria passim* being abundant in co-located honey bees.

#### 3.2.5. Unidentified Diseases

In addition to the studies discussed above, we found six reports of stingless bees developing apparent disease symptoms in cases where the causal agent could not be conclusively identified. Five studies discussed a syndrome that manifested as a seasonal collapse of colonies, while the sixth examined symptoms that superficially resembled DWV.

Since 2014, stingless beekeepers have reported a regular seasonal collapse of managed colonies of *M. quadrifasciata* in the Brazilian state of Rio Grande do Sul, an area to which this species is native, but where it is now believed to be extinct outside managed colonies [121]. Each year, from February to April, workers in some hives lose the ability to fly and suffer from tremors and paralysis, after which their colonies often collapse [122,123]. This effect is commonly referred to as the ‘annual syndrome’ or ‘evil of March disease’.

Diaz et al. [122] investigated the annual syndrome using DNA metabarcoding of bees from both affected and apparently healthy colonies to determine if the annual syndrome was caused by any known pathogenic bacteria. They failed to find any known bee pathogens and ruled out toxicity from foraged material but were unable to determine a specific cause. Similarly, Caesar et al. [123] used metabarcoding techniques to examine the virome of colonies suffering from the annual syndrome, along with apparently healthy colonies, and compared their findings to a database of known viruses. They found higher viral loads in the affected colonies but could not directly link any specific virus to incidences of the syndrome and suggested that the detected viruses were more likely to be co-stressors associated with weakened hives than the causal agents of colony collapse.

Haag et al. [124] also investigated the annual syndrome by examining the gut microbiome and foraging behavior of *M. quadrifasciata* throughout the year and found both changed substantially over the seasons. Diet became substantially less diverse between January and February (when the annual syndrome begins to manifest), becoming dominated by exotic *Eucalyptus* pollen that is lower in fatty acids than the native pollen that dominates *M. quadrifasciata*’s diet during other times of the year. This was accompanied by reductions in the diversity of bacteria in the bees’ core gut microbiome. The authors suggested that these changes are likely stressors that contribute to a colony becoming vulnerable to the annual syndrome. However, as with the other studies discussed above, Haag et al. [124] were unable to identify a single causal agent. A follow up study by Caesar and Haag [125] of bacteriophages present within these same bees revealed that phages of the class Caudoviricetes were only present in bees exhibiting symptoms of the annual syndrome. The authors suggested this may indicate undetected differences in the gut microbiome that may have a causal relationship with the syndrome but were unable to elucidate any further details.

Caesar et al. [121] concluded that the annual syndrome likely has both a genetic and an environmental component after raising pairs of mother–daughter hives in geographically distant locations. They found that two mother hives in one location were severely impacted by the annual syndrome, while their daughter hives in another location were mildly affected and a third mother–daughter pair in the same locations showed no apparent symptoms. They also ruled out lethal toxic impacts, failing to find detectable levels of any of 197 known toxic compounds in bees from the affected colonies. All five studies that examined the annual syndrome were unable to unambiguously define its cause, with most suggesting it was the result of the cumulative impact of multiple sub-lethal factors.

The sixth study examining a symptomatic disease with unknown cause was that of Al Toufailia et al. [126]. The authors observed a colony of the Brazilian species *Scaptotrigona depilis* exhibiting symptoms consistent with DWV (adults with shriveled wings and an inability to fly) and noted that the affected colony seemed to show poorer hygienic ability than other colonies (i.e., fewer diseased larvae were removed). However, they were unable to identify the cause, with genetic analysis showing no evidence of the virus known to cause DWV in honey bees.

### 3.3. Overview of Stingless Bee Pests and Diseases

The above review illustrates two main knowledge gaps regarding pests and diseases of stingless bees. The first is that the literature likely underestimates the number of pests and diseases that affect meliponines. The second is that relationships between the presence of potential pests/pathogens and negative impacts on stingless bees are often unclear.

The likely underestimation of the number of pests and diseases able to impact stingless bees is illustrated by the substantial difference in the number of studies reporting on these compared to honey bees. While managed honey bees consist primarily of a single species, compared to an estimated 500 species of stingless bees in numerous genera, most of our initial examinations of the literature returned at least an order of magnitude more results for searches related to honey bees than to stingless bees (Table S3).

While it is possible that this could be a result of stingless bees being far less vulnerable to pests and diseases than honey bees, the only work that has compared pest and disease levels between stingless bees and honey bees provides evidence against this. The survey by Dias de Freitas et al. [17] found that both stingless bees and honey bees experience pest and disease attacks at similar levels—85% and 77% of honey bee and stingless bee keepers respectively reported pests impacting their hives, while 64% and 67% reported disease impacts. While Dias de Freitas et al. [17]’s work was limited to Brazil, meaning caution must be exercised in extrapolating its findings more broadly, it raises the likelihood that the large difference in the number of studies represents a bias in research carried out. The much larger size of the apiculture industry compared to the meliponiculture industry [127,128] provides logical support to the idea that honey bees would be much more well-researched.

Furthermore, even if stingless bees are truly less vulnerable to pests and diseases than honey bees, this situation may be an artefact of the different levels of domestication of the two groups. Honey bees have been domesticated for millennia. This domestication has increased the expression of traits valuable to humans; however, it is likely that selective breeding for these traits has resulted in some loss of the natural ability to resist diseases and pests [129]. Aside from a small number of species from Central and South America, stingless bees have only been domesticated for a few decades—if their use is to be expanded, this may also be accompanied by reduced fitness, and thus the situation regarding pests and diseases that honey bees currently face may serve as a warning of what a future meliponiculture industry may have to deal with.

While this review was intended to analyze the research literature, comparing our findings with the popular literature also supports the theory that stingless bee pests and diseases are under-researched. For example, two popular guides to stingless beekeeping in Australia, Heard [6] and Klumpp [130], both list the hoverfly *Ceriania ornata* (Diptera: Syrphidae) as one of the most significant pests of stingless bees in that country. However, we found no primary studies analyzing the interaction of this fly with stingless bees.

An inverse situation to this may also exist regarding novel pests and those that have a major impact on honey bees. SHBs and mites were amongst the most researched pests; however, the summation of the findings is that their impact on healthy stingless bee colonies appears to be relatively minor. Both were likely the subject of a disproportionately high level of research as a result of their major impacts on honey bees and, in the case of SHB, the fact that it is a newly arrived pest in many parts of the world, spurring additional research interest.

In addition to the knowledge gap regarding the number of studies conducted, a second gap is that little is known about the likely impacts of many of the pests and diseases studied. While the damage caused by some better documented pests or diseases, such as cleptoparasitic bees and phorid flies, is well-established, this is not the case for other groups. In many situations, it is unclear if the presence/detection of a putative pest or disease in an unhealthy colony is the cause of this condition or a symptom of it.

This uncertainty may be attributable, in part, to the large number of ‘case study’ research papers found. Rather than being part of a systematic research program, these papers were typically produced after researchers fortuitously became aware of problems in one or more colonies and/or the presence of a potential pest or disease. While such studies can provide valuable information, they typically do not allow the circumstances that preceded the arrival of the pest or pathogen, including the health of the colony at that time, to be accurately measured.

Specifically regarding stingless bee diseases, while numerous studies found known pathogens (mostly of honey bees) on or in stingless bees or their hives, few established a link with specific disease symptoms. Such connections may be difficult to establish, as many significant honey bee diseases are asymptomatic in adult workers, primarily impacting brood or queens. With such diseases, confirmation using Koch’s postulates requires detailed hive-level studies, which can be complex and expensive to carry out at scale.

## 4. Conclusions

Much of the research to date on pests and diseases of managed bees has focused on honey bees, likely due to their large global spread and economic importance. However, with the need to diversify managed pollinators due to the range of threats to honey bees, the global importance of the stingless beekeeping industry is likely to expand and, as a result, the impacts of pests and diseases on stingless bees are likely to become more apparent. This will probably increase as stingless bees are subjected to higher levels of domestication, selection, and management for productive activities, including pollination.

## Figures and Tables

**Table 1:** Search terms employed.

Search Number	Search Terms
1	pest OR parasit* OR predat*
2	diseas* OR virus* OR pathogen* OR bacter* OR fung*
3	“small hive beetle” OR “Aethina tumida”
4	phorid OR Phoridae OR Dohrniphora
5	Ceriana OR syrph* OR hoverfl* OR “hover fly” OR “hover flies”
6	Syntretus
7	“wax moth” OR “wax moths” OR Galleria OR Achroia
8	Nosema
9	foulbrood OR “foul brood”
10	Hyg*
11	mite* OR Acari
12	wasp* OR Bembix
13	Hermetia OR “soldier fly” OR “soldier flies”
14	Mantispid* OR mantisfly OR mantisflies OR “mantis fly” OR “mantis flies”
15	cleptoparas* OR kleptoparas* OR cleptobios* OR kleptobios* OR robb* OR usurp* OR Lestrimelitta OR Cleptotrigona

* Represents truncation, so the database returns all forms of that word.

**Table 2:** Inclusion/exclusion criteria for documents included in review.

Criterion Number		Inclusion Criteria	Exclusion Criteria
1	Subject Organism	Stingless bees.	
2	Data Type	Primary data not previously reported elsewhere.	
3	Relationship to Stingless Bees	Document examined a potential pest or disease for its relationship to stingless bee colonies (including examinations of whether pests or diseases harmful to other organisms can affect stingless bees).	Document only examined beneficial or commensal organisms, without evaluating their potential to serve as pests.
4a	Impact (Pests)	Document examined an organism known or suspected to affect stingless bee colonies and place them at risk of collapse, through specialising in invading hives or reproducing within hives, and investigated its impact in some way. Studies were included even if they came to the conclusion that an organism suspected to be harmful was not indeed harmful, or were inconclusive in this regard.	Document only examined organisms able to harm individual stingless bees outside hives (e.g., predators such as spiders and birds). Generalist predators that may occasionally destroy hives but do not specialise in doing so or do not reproduce within hives (e.g., ants, chimpanzees) were excluded. Studies that only used simulated pests without using these simulations to investigate hive level impacts were excluded.
4b	Impact (Disease)	Document examined a pathogen or symptom known or suspected to reproduce within stingless bee colonies and place them at risk of collapse.	Document only examined commensal microbes. Documents that examined chemistry or microbiota of hives or hive products were excluded unless the effect on stingless bee disease was examined.
5	Document Type	Peer-reviewed publications or theses.	Other document types were excluded as they typically did not systematically report on primary research findings in a manner that allowed findings to be replicated. Where a thesis and a published paper both reported on the same data set, the thesis was excluded.
6	Geographic Area	All	
7	Age of Document	All	
8	Language	Document published in English, Spanish, Portuguese or French	

**Table 3:** Summary of pest studies found.

Pest Group	Number of Studies	Locations Reported	Associated Impacts
Cleptoparasitic bees (stingless bees from numerous facultative and obligate cleptoparasitic species)	10	Angola, Australia, Brazil, Panama	Resource robbing—removal of hive resources. Usurpation—entire hive population killed and replaced by offspring of attacking hive.
Small hive beetle *(Aethina tumida*; Coleoptera, Nitidulidae)	9	Australia, Brazil, Cuba, Kenya, Mexico, Philippines (single species native to sub-Saharan Africa, invasive in other regions)	Larvae feed on hive provisions and brood, waste ferments in hive. Associated with hive collapse.
Other beetles (various species of order Coleoptera; families Nitidulidae and Histeridae)	6	Brazil, Indonesia, Malaysia, Thailand	Larvae and adults feed on hive provisions and brood, waste ferments in hive. Associated with hive collapse.
Mites (various species of order Acari, families Acaraidae, Carpoglyphidae, Erythraeidae, Pyemotidae and Phytoseiidae)	9	Argentina, Brazil, Colombia, India, Mexico	Feeding on hive provisions, brood and adult bee tissues and body fluids. Possible vectoring of disease. Associated with hive collapse.
Phorid flies (various species of order Diptera, family Phoridae)	8	Australia, Brazil, Colombia, Costa Rica, El Salvador, Nicaragua	Endoparasitism of adult bees (primarily drones). Larvae and adults feed on hive provisions and brood, waste ferments in hive. Associated with hive collapse.
Soldier flies (*Hermetia illucens* and *Hermetia fenestrate*, Diptera; Stratiomyidae)	4	Brazil, India, Malaysia	Feeding on hive detritus and brood. Associated with hive collapse.
Wax moths (*Galleria mellonella* and *Achroia grisella*, Lepidoptera; Pyralidae)	2	Brazil, India	Feeding on hive detritus and brood.
Mantisflies (*Plega hagenella*, Neuroptera; Mantispidae)	1	Brazil	Feeding on brood.

Total number of studies exceeds 48, as some studies examined multiple types of pests. In many cases, it is unclear whether hive collapse has a causal or merely correlative relationship with a particular pest group—this is discussed further in the text.

**Table 4:** Studies on viruses of stingless bees.

Study	Location	Bee Species Examined ^1^	Virus Presence							Virus Replication	Symptoms Present
			DWV	BQCV	ABPV	CBPV	SBV	IBPV	KBV		
Teixeira et al. [98]	Brazil	MMa	-	-	-	-	NA	-	-	NA	NA
		MQ	-	-	-	-	NA	-	-	NA	NA
		MMS	-	-	-	-	NA	-	-	NA	NA
		MCm	-	+	-	+	NA	-	-	NA	NA
		MRR	-	-	-	-	NA	-	-	NA	NA
		MRM	-	+	-	-	NA	-	-	NA	NA
Alvarez et al. [110]	Argentina	TrS	-	-	-	-	-	+	NA	NA	NA
		TeF	+	-	-	-	-	+	NA	NA	NA
		TeCl	-	-	-	-	-	-	NA	NA	NA
		LC	-	-	-	-	-	-	NA	NA	NA
		PE	-	-	+	-	-	+	NA	NA	NA
		PD	-	-	+	-	-	+	NA	NA	NA
		SP	-	-	-	-	-	-	NA	NA	NA
		SD	-	-	-	-	-	-	NA	NA	NA
		SQ	-	-	-	-	-	-	NA	NA	NA
		CC	-	-	-	-	-	-	NA	NA	NA
		MT	-	-	-	-	-	-	NA	NA	NA
		MQ	-	-	-	-	-	-	NA	NA	NA
de Souza et al. [111]	Brazil	MSu	+	NA	NA	NA	NA	NA	NA	NA	NA
Fleites-Ayil et al. [112]	Mexico	MB	+	+	NA	NA	NA	NA	NA	Replication observed	Possible symptoms observed
Guzman-Novoa et al. [113]	Mexico	SM	+	+	-	-	-	-	NA	Replication observed	NA
Morfin et al. [114]	Mexico	MCl	+	+	NA	NA	NA	NA	NA	Replication observed	NA
Oliveira França et al. [115]	Brazil	MSc	-	-	+	-	NA	-	NA	NA	NA
		MQ	-	-	+	-	NA	+	NA	NA	NA
		NT	-	-	+	-	NA	-	NA	NA	NA
		TeA	-	-	+	-	NA	+	NA	NA	NA
		SX	-	-	+	-	NA	-	NA	NA	NA
		PHe	-	-	+	-	NA	-	NA	NA	NA
		TrS	-	-	+	-	NA	-	NA	NA	NA
Tapia-Gonzalez et al. [116]	Mexico	MCl	+	+	-	-	-	-	NA	Replication observed (DWV and BQCV)	NA
		TrF	+	-	-	-	-	-	NA	Replication observed (DWV)	NA
		NP	+	-	-	-	-	-	NA	Replication observed (DWV)	NA
		SM	+	+	-	-	-	-	NA	No replication observed	NA
Ueira-Vieira et al. [117]	Brazil	MSc	-	-	+	-	-	-	-	NA	Possible symptoms observed
Zhang et al. [118]	China	TeL	+	-	-	-	-	-	NA	NA	NA
Amirthalingam et al. [99]	Malaysia	HI	-	-	-	-	-	NA	NA	NA	NA
		GT	-	-	-	-	-	NA	NA	NA	NA
Cristina Dias et al. [102]	Brazil	FV	+	+	+	-	NA	-	-	NA	NA
Guimaraes-Cestaro et al. [106]	Brazil	NT	+	+	+	-	NA	+	+	NA	NA
		TeA	+	+	+	-	NA	+	+	NA	NA
		TeE	+	+	+	+	NA	+	+	NA	NA
Roy [97]	Australia	TeCa	NA	+	NA	NA	-	NA	NA	NA	NA
		TeH	NA	-	NA	NA	-	NA	NA	NA	NA
		AA	NA	-	NA	NA	NA	NA	NA	NA	NA
Nkoba et al. [103]	Kenya	HG	-	+	-	-	-	-	-	NA	NA
		MB	-	+	-	-	-	-	-	NA	NA
		MF	-	-	-	-	-	-	-	NA	NA
		MTg	-	+	-	-	-	-	-	NA	NA
		MBe	-	-	-	-	-	-	-	NA	NA
		ML	-	+	-	-	-	-	-	NA	NA
		DS	-	-	-	-	-	-	-	NA	NA
		Li. spp.	-	-	-	-	-	-	-	NA	NA
		PHi	-	-	-	-	-	-	-	NA	NA
Brettell et al. [104]	Australia	TeCa	+	NA	NA	NA	+	-	NA	NA	NA

Explanation of symbols: ‘-’ virus not detected, ‘+’ virus detected, ‘NA’ virus presence, replication or symptoms not assessed. ^1^ Species abbreviations: AA: *Austroplebia australis,* DS: *Dactylurina schmidt;* FV: *Frieseomelitta varia*; GT: *Geniotrigona thoracica*; HG: *Hypotrigona gribodoi*; HI: *Heterotrigona itama*; LC: *Lestrimelitta chacoana*; Li. spp.: *Liotrigona* spp.; MB: *Melipona beecheeii*; MCm: *Melipona compressipes*; MCl: *Melipona colimana*; MMa: *Melipona marginata*; MMS: *Melipona mandacaia*; MQ: *Melipona quadrifasciata*; MRM: *Melipona mondury*; MRR: *Melipona rufiventris*; MSu: *Melipona subnitida*; MSc: *Melipona scutellaris*; MT: *Melipona torrida*; MB: *Meliponula bocandei*; MF: *Meliponula ferruginea*; MTg: *Meliponula togoensis*; MBe: *Meliponula beccarii*; ML: *Meliponula lendiliana*; NP: *Nannotrigona perilapoides*; NT: *Nannotrigona testaceicornis*; PD: *Plebeia droryana*; PE: *Plebeia emerinoides*; PHe: *Partamona helleri*; PHi: *Plebeina hildebranti*; SD: *Scaptotrigona depilis*; SM: *Scaptotrigona Mexicana*; SP: *Scaptotrigona aff. postica*; SQ: *Schwarziana quadripunctata*; SX: *Scaptotrigona xanthotricha*; TeA: *Tetragonisca angustula*; TeCa: *Tetragonula carbonaria*; TeCl: *Tetragona clavipes*; TeE: *Tetragona elongata*; TeF: *Tetragonisca fiebrigi*; TeH: *Tetragonula hockingsi*; TeL: *Tetragonula laeviceps*; TrF: *Trigona fulviventris*; TrS: *Trigona spinipes*.

## Data Availability

All relevant data are contained either within the main text or Supplementary Materials section of this manuscript.

## Supplementary Materials

The following supporting information can be downloaded at: https://www.mdpi.com/article/10.3390/insects17060619/s1, Table S1: Translation of Search Terms; Table S2: Full List of Included Studies; Table S3: Initial Search Results.
