# Supplementary material for: A Global Review of the Pests and Diseases of Stingless Bees
Source: Insects. 2026 Jun 11;17(6):619. doi: 10.3390/insects17060619 (PMC13299999; doi:10.3390/insects17060619)
Supplement: Supplementary file 1 [file insects-17-00619-s001.zip › insects-4200519-supplementary.pdf]

Supporting Information for “A Global Review of the Pests and Diseases of Stingless Bees”

S1 – Translation of Search Terms

Each search string in Tables S1A and S1B is the translation of the corresponding English language search terms in main text Table 1.

All searches in Spanish contained the following search string:

“abeja sin aguijón” OR "abejas sin aguijón" OR melipon*

In addition to the one of the search strings listed in Table S1A.

Table S1A Spanish Translations of Search Terms

Search Number	Search Terms
1	parásit* OR depred*
2	enferm* OR virus OR patógen* OR bacterias OR hongo*
3	“aethina tumida” OR "pequeño escarabajo de la colmena"
4	phorid OR phoridae OR Dohrniphora
5	Cerania OR syrph* OR sírfido* OR "mosca flotante"
6	Syntretus
7	polilla de la cera OR "polillas de cera" OR Galleria OR Achroia
8	Nosema
9	loque
10	higien*
11	ácaro* OR acari
12	avispa* OR bembix
13	"moscas soldado" OR "mosca soldado" OR Hermetia

14	Mantispid* OR "mosca mantis" OR "moscas mantis"
15	cleptoparas* OR kleptoparas* OR cleptobios* OR kleptobios* OR robo* OR roba OR usurp* OR Lestrimelitta OR Cleptotrigona

All searches in Portuguese contained the following search string:

“abelha sem ferrão” OR "abelhas sem ferrão" OR melipon*

In addition to the one of the search strings listed in Table S1B.

**Table S1B** Portuguese Translations of Search Terms	
Search Number	Search Terms
1	praga* OR parasit* OR predador*
2	doen* OR virus OR patogen* OR bactéria* OR fung*
3	"aethina tumida" OR "pequeno besouro da colmeia"
4	phorid OR phoridae OR Dohrniphora
5	mosca-das-flores OR moscas-das-flores OR Cerania OR syph*
6	Syntretus
7	traça da cera OR "traças da cera" OR Galleria OR Achroia
8	Nosema
9	cria pútrida OR "ninhada imunda"
10	higiên*
11	ácaro* OR acari
12	vespa* OR bembix
13	"moscas soldado" OR "mosca soldado" OR Hermetia
14	mosca-louva-a-deus OR moscas-louva-a-deus OR Mantispid*
15	cleptoparas* OR kleptoparas* OR cleptobios* OR kleptobios* OR roub* OR usurp* OR Lestrimelitta OR Cleptotrigona

Table S2 – Full List of Included Studies

Article	Source[1]	Publication Type	Study Type[2]	Study Location	Bee Species Studied	Pest or Disease Studied[3]
Al Toufailia, H., Alves, D. A., Bento, J. M., Marchini, L. C., & Ratnieks, F. L. (2016). Hygienic behaviour in Brazilian stingless bees. *Biol Open*, *5*(11), 1712-1718. https://doi.org/10.1242/bio.018549	Search 1	Journal Article	Systematic manipulation	Brazil	*Melipona scutellaris, Scaptotrigona depilis, Tetragonisca angustula*	Unknown disease with symptoms superficially resembling DWV
Alvarez, L. J., Reynaldi, F. J., Ramello, P. J., Garcia, M. L. G., Sguazza, G. H., Abrahamovich, A. H., & Lucia, M. (2017). Detection of honey bee viruses in Argentinian stingless bees (Hymenoptera: Apidae). Insectes Sociaux, 65(1), 191-197. https://doi.org/10.1007/s00040-017-0587-2	Search 2	Journal Article	Systematic observation	Argentina	*Trigona spinipes, Tetragonisca fiebrigi, Tetragona clavipes, Tetragonisca fiebrigi, Lestrimelitta chacoana, Plebeia emerinoides, Plebeia droryana, Scaptotrigona aff. Postica, Scaptotrigona depilis, Schwarziana quadripunctata*	DWV, BQCV, APPV, CBPV, SBV, IBPV, KBV
Amirthalingam, V., Ataa, N. S. M., Mahmuddin, S. S., Adam, N. A., Nai, Y.-S., & Lau, W.-H. (2024). First record of Aspergillus caelatus as a pathogen of Heterotrigona itama and Geniotrigona thoracica in Malaysia. *Journal of Asia-Pacific Entomology*, *27*(1). https://doi.org/10.1016/j.aspen.2023.102173	Search 1	Journal Article	Systematic manipulation, Systematic observation	Malaysia	*Heterotrigona itama, Geniotrigona thoracica*	WV, BQCV, APPV, CBPV, SBV, IBPV, KBV, American Foulbrood, European Foulbrood, Shanks Brood Disease, *Aspergillus caelatus*

Article	Source[1]	Publication Type	Study Type[2]	Study Location	Bee Species Studied	Pest or Disease Studied[3]
Bassini-Silva, R., de Souza, B. A. G. T., Welbourn, C., Barros-Battesti, D. M., & Jacinavicius, F. d. C. (2024). First host-association record for Leptus (Leptus) adaminae Haitilinger, 2004 (Trombidiformes: Erythraeidae). *Biologia*, *80*(3), 655-660. https://doi.org/10.1007/s11756-024-01855-7	Search 1	Journal Article	Case study	Brazil	*Melipona quadrifasciata*	Mites (*Leptus adaminae*)
Bobadoye, B. O., Fombong, A. T., Kiatoko, N., Suresh, R., Teal, P. E. A., Salifu, D., & Torto, B. (2018). Behavioral responses of the small hive beetle, Aethina tumida, to odors of three meliponine bee species and honey bees, Apis mellifera scutellata. *Entomologia Experimentalis et Applicata*, *166*(7), 528-534. https://doi.org/10.1111/eea.12700	Search 1	Journal Article	Systematic manipulation	Kenya	*Meliponula bocandei, Meliponula ferruginea.*	Small Hive Beetle (*Aethina tumida*)
Brettell, L. E., Riegler, M., O'Brien, C., & Cook, J. M. (2020). Occurrence of honey bee-associated pathogens in Varroa-free pollinator communities. J Invertebr Pathol, 171, 107344. https://doi.org/10.1016/j.jip.2020.107344	Reference Search	Journal Article	Systematic observation	Australia	*Tetragonula carbonaria*	DWV, SBV, IBPV, Lake Sinai Virus
Brown, B. V. (1997a). Parasitic Phorid Flies: A Previously Unrecognised Cost to Aggregation Behavior of Male Stingless Bees. *Biotropica*, *29*(3), 370-372.	Search 1	Journal Article	Case study	Costa Rica	*Cephalotrigona capitata*	Phorids (*Apocephalus apivorus*)
Caesar, L., Cibulski, S. P., Canal, C. W., Blochtein, B., Sattler, A., & Haag, K. L. (2019). The virome of an endangered stingless bee suffering from annual mortality in southern Brazil. J Gen Virol, 100(7), 1153-1164. https://doi.org/10.1099/jgv.0.001273	Search 2	Journal Article	Systematic observation	Brazil	*Melipona quadrifasciata*	Unknown "annual syndrome" disease
Caesar, L., & Haag, K. L. (2024). Tailed bacteriophages (Caudoviricetes) dominate the microbiome of a diseased stingless bee. Genet Mol	Search 2	Journal Article	Systematic observation	Brazil	*Melipona quadrifasciata*	Unknown "annual syndrome" disease

Article	Source[1]	Publication Type	Study Type[2]	Study Location	Bee Species Studied	Pest or Disease Studied[3]
Biol, 46(3 Suppl 1), e20230120. https://doi.org/10.1590/1678-4685-GMB-2023-0120						
Caesar, L., Lopes, A. M. C., Radaeski, J. N., Bauermann, S. G., Konzen, E. R., Pombert, J.-F., Sattler, A., Blochtein, B., Carvalho, A. T., & Haag, K. L. (2021). Longitudinal survey reveals delayed effects of low gene expression on stingless bee colony health. Journal of Apicultural Research, 61(5), 654-663. https://doi.org/10.1080/00218839.2021.1962123	Search 2	Journal Article	Systematic observation	Brazil	*Melipona quadrifasciata*	Unknown "annual syndrome" disease
Camenzind, D. W., Chanasit, W., Germann, C., Yañez, O., Neumann, P., Maitip, J., & Straub, L. (2024). A novel parasitic beetle, Procoryphaeus violaceus, in stingless bees, Tetragonula pagdeni. *Journal of Apicultural Research*, *63*(4), 688-690. https://doi.org/10.1080/00218839.2024.2348416	Search 1	Journal Article	Case study	Thailand	*Tetragonula pagdeni*	Beetles (*Procoryphaeus violaceus*)
Carvalho, A. F., Leivas, F. W. T., & Souza, T. B. (2021). Feeding Habits and Behavior of a Bee Killer: Hololepta reichii (Coleoptera, Histeridae). *Neotrop Entomol*, *50*(2), 317-320. https://doi.org/10.1007/s13744-020-00818-2	Search 1	Journal Article	Case study, Systematic manipulation	Brazil	*Scaptotrigona xanthotricha*	Beetles (Clown beetle - *Hololepta reichii*)
Cepeda-Aponte, O. I., Imperatriz-Fonseca, V. L., & Velthuis, H. H. W. (2002). Lesser Wax MothAchroia Grisella:First Report for Stingless Bees and New Capture Method. *Journal of Apicultural Research*, *41*(3-4), 107-108. https://doi.org/10.1080/00218839.2002.11101077	Search 1	Journal Article	Case study	Brazil	*Melipona bicolor bicolor, Melipona quadrifasciata*	Wax Moths (*Achroia grisella*)
Cervancia, C. R., de Guzman, L. I., Polintan, E. A., Dupo, A. L. B., & Locsin, A. A. (2016). Current status of small hive beetle infestation in the Philippines.	Search 3	Journal Article	Case Study	Philippines	*Tetragonula sp.*	Small Hive Beetle (*Aethina tumida*)

Article	Source[1]	Publication Type	Study Type[2]	Study Location	Bee Species Studied	Pest or Disease Studied[3]
Journal of Apicultural Research, 55(1), 74-77. https://doi.org/10.1080/00218839.2016.1194053						
Correia, C. S., da Silva Melo, É. C., Navarro, D. M. D. A. F., Carvalho, A. T., & Maia, A. C. D. (2026). Under the radar: the kleptoparasite Pseudohypocera kerteszi evades meliponine bee defense without cuticular hydrocarbon mimicry or camouflage: CS Correia et al. *Apidologie*, *57*(2), 15. https://doi.org/10.1007/s13592-026-01258-4	Search 15	Journal Article	Systematic manipulation	Brazil	*Scaptotrigona tubiba*	Phorid flies (*Pseudohypocera kerteszi*)
Costa, L., Galaschi-Teixeira, J. S., Maia, U. M., & Imperatriz-Fonseca, V. L. (2018). Plasticity of stingless bee Melipona fuliginosa Lepeletier to obtain food resources in Amazonia. *Sociobiology*, *65*(4), 744-750.	Search 15	Journal Article	Case study	Brazil	*Melipona paraensis, Melipona fasciculata*	Facultatively cleptoparasitic stingless bees (*Melipona fuliginosa*)
Cristina Dias, A., Taís Ferreira, J., Weinstein Teixeira, É., & Pedro Lourenço, A. (2023). Honey bee viruses in solitary bees in South America: simultaneous detection and prevalence. Journal of Apicultural Research, 63(1), 122-127. https://doi.org/10.1080/00218839.2023.2190066	Search 2	Journal Article	Systematic observation	Brazil	*Frieseomelitta varia*	Nosema, DWV, BQCV, APPV, CBPV, IBPV, KBV
Cunningham, J. P., Hereward, J. P., Heard, T. A., De Barro, P. J., & West, S. A. (2014). Bees at war: interspecific battles and nest usurpation in stingless bees. *The American Naturalist*, *184*(6), 777-786.	Search 15	Journal Article	Case study, Systematic Observation	Australia	*Tetragonula carbonaria*	Usurpation by other bees (*Tetragonula hockingsi*)
da Silva, J. A., Jantsch Ferla, J., Pallini, A., Barchuk, A. R., & Wolowski, M. (2022). First report of Tyrophagus putrescentiae (Schrank) (Acari: Acaridae) in colonies of the stingless bee Frieseomelitta varia (Hymenoptera, Apidae, Meliponini). *Journal of Apicultural Research*, *62*(5), 1166-1168. https://doi.org/10.1080/00218839.2022.2034215	Search 1	Journal Article	Case study	Brazil	*Frieseomelitta varia*	Mites (*Tyrophagus putrescentiae*)

Article	Source[1]	Publication Type	Study Type[2]	Study Location	Bee Species Studied	Pest or Disease Studied[3]
de Portugal-Araújo, V. (1958). A contribution to the bionomics of Lestrimelitta cubiceps (Hymenoptera, Apidae). *Journal of the Kansas Entomological Society*, *31*(3), 203-211.	Reference search	Journal Article	Systematic manipulation	Angola	*Trigona braunsi*	Obligate cleptoparasitic bees (*Cleptotrigona cubiceps*)
de Souza, F. S., Kevill, J. L., Correia-Oliveira, M. E., de Carvalho, C. A. L., & Martin, S. J. (2019). Occurrence of deformed wing virus variants in the stingless bee Melipona subnitida and honey bee Apis mellifera populations in Brazil. J Gen Virol, 100(2), 289-294. https://doi.org/10.1099/jgv.0.001206	Search 1	Journal Article	Systematic observation	Brazil	*Melipona subnitida*	DWV
Depintor, T. S., & De Jong, D. (2025). Scientific note on mortality of a stingless bee Tetragona clavipes colony infested with clown beetles Hololepta reichii (Histeridae) and black soldier flies Hermetia illucens (Stratiomyidae) in Southeastern Brazil. Apidologie, 56(1). https://doi.org/10.1007/s13592-024-01142-z	Search 13	Journal Article	Case Study	Brazil	*Tetragona clavipes*	Clown Beetles (*Hololepta reichii*), Soldier Flies (*Hermetia illucens*)
Devanesan, S., Nisha, M. M., Shailaja, K. K., & Bennet, R. (2003). Natural Enemies of the Stingless Bee Trigona iridipennis Smith in Kerala. Insect Environment, 9(1).	Reference Search	Journal Article	Case Study	India	*Trigona iridipennis*	Mites (*Amblyseius* sp.), Soldier Flies (*Hermetia illucens*)
Dias de Freitas, C., Oki, Y., Resende, F. M., Zamudio, F., Simone de Freitas, G., Moreira de Rezende, K., Amaro de Souza, F., De Jong, D., Quesada, M., Siqueira Carvalho, A., Silvia Soares Pires, C., & Fernandes, G. W. (2022). Impacts of pests and diseases on the decline of managed bees in Brazil: a beekeeper perspective. *Journal of Apicultural Research*, *62*(5), 969-982. https://doi.org/10.1080/00218839.2022.2099188	Search 1	Journal Article	Systematic observation	Brazil	various	Various (survey of all beekeepers about all pest and diseases encountered). **Note:** This was the only study found that examined both pests and diseases and is thus included in

Article	Source[1]	Publication Type	Study Type[2]	Study Location	Bee Species Studied	Pest or Disease Studied[3]
						the count total for both.
Diaz, S., de Souza Urbano, S., Caesar, L., Blochtein, B., Sattler, A., Zuge, V., & Haag, K. L. (2017). Report on the microbiota of Melipona quadrifasciata affected by a recurrent disease. J Invertebr Pathol, 143, 35-39. https://doi.org/10.1016/j.jip.2016.11.012	Search 2	Journal Article	Systematic observation	Brazil	*Melipona quadrifasciata*	Unknown "annual syndrome" disease
Disney, R. H. L., & Bartareau, T. (1995). A new species of Dohrniphora (Diptera: Phoridae) associated with a stingless bee (Hymenoptera: Apidae) in Australia. Sociobiology, 26(3), 229-239.	Reference Search	Journal Article	Systematic observation	Australia, Brazil, Nicaragua	*Tetragonula carbonaria*	Phorids (*Dorniphora trigonae*, *Pseudohypocera kerteszi*)
Fleites-Ayil, F. A., Medina-Medina, L. A., Quezada Euán, J. J. G., Stolle, E., Theodorou, P., Tragust, S., & Paxton, R. J. (2023). Trouble in the tropics: Pathogen spillover is a threat for native stingless bees. Biological Conservation, 284. https://doi.org/10.1016/j.biocon.2023.110150	Search 2	Journal Article	Systematic manipulation	Mexico	*Melipona beecheeii*	DWV, BQCV
Gopinatha, B. N., & Basavarajappa, S. (2022). Pests and predators interference on the stingless bee population inhabited at different habitats amidst south-eastern Karnataka, India. *weather*, *41*(42), 43-44.	Search 15	Journal Article	Systematic observation	India	Numerous unspecified species.	Wax Moths (*Galleria mellonella)*
Greco, M. K., Hoffmann, D., Dollin, A., Duncan, M., Spooner-Hart, R., & Neumann, P. (2010). The alternative Pharaoh approach: stingless bees mummify beetle parasites alive. Naturwissenschaften, 97(3), 319-323. https://doi.org/10.1007/s00114-009-0631-9	Search 1	Journal Article	Systematic manipulation	Australia	*Tetragonulla carbonaria*	Small Hive Beetle (*Athina tumida*)
Guimaraes-Cestaro, L., Martins, M. F., Martinez, L. C., Alves, M., Guidugli-Lazzarini, K. R., Nocelli, R. C. F., Malaspina, O., Serrao, J. E., & Teixeira, E. W. (2020). Occurrence of virus, microsporidia, and	Search 1	Journal Article	Systematic observation	Brazil	*Nannotrigona testaceicornis, Tetragonisca*	Nosema, DWV, BQCV, APPV, CBPV, IBPV, KBV

Article	Source[1]	Publication Type	Study Type[2]	Study Location	Bee Species Studied	Pest or Disease Studied[3]
pesticide residues in three species of stingless bees (Apidae: Meliponini) in the field. Naturwissenschaften, 107(3), 16. https://doi.org/10.1007/s00114-020-1670-5					*angustula, Tetragona elongata*	
Guzman-Novoa, E., Hamiduzzaman, M. M., Anguiano-Baez, R., Correa-Benítez, A., Castañeda-Cervantes, E., & Arnold, N. I. (2016). First detection of honey bee viruses in stingless bees in North America. Journal of Apicultural Research, 54(2), 93-95. https://doi.org/10.1080/00218839.2015.1100154	Search 2	Journal Article	Systematic observation	Mexico	*Scaptotrigona Mexicana*	DWV, BQCV, APPV, CBPV, SBV, IBPV, KBV
Haag, K. L., Caesar, L., Regueira-Neto, M. d. S., de Sousa, D. R., Marcelino, V. M., Balbino, V. d. Q., & Carvalho, A. T. (2022). Temporal changes in gut microbiota composition and pollen diet associated with colony weakness of a stingless bee. Microb. Ecol. 85, 1514–1526 https://doi.org/10.21203/rs.3.rs-1254847/v1	Search 2	Journal Article	Systematic observation	Brazil	*Melipona quadrifasciata*	Unknown “annual syndrome” disease
Halcroft, M., Spooner-Hart, R., & Neumann, P. (2010). Behavioral defense strategies of the stingless bee, Austroplebeia australis, against the small hive beetle, Aethina tumida. Insectes Sociaux, 58(2), 245-253. https://doi.org/10.1007/s00040-010-0142-x	Search 1	Journal Article	Systematic manipulation	Australia	*Austroplebeia australis*	Small Hive Beetle (*Aethina tumida*)
Hashim, N. A., Bahri, A. R. S., Basari, N., & Sharudin, N. H. (2017). Mass infestation of black soldier fly Hermetia illucens (Diptera: Stratiomyidae) on colonies of the Indo-Malayan stingless bees Geniotrigona thoracica and Heterotrigona itama. Journal of Biodiversity and Envrionmental Science, 11(2), 9-15.	Reference Search	Journal Article	Case Study, Systematic observation	Malaysia	*Geniotrigona thoracica, Heterotrigona itama*	Soldier Flies (*Hermetia illucens*)
Inoue, T., Nakamura, K., Salmah, S., & Abbas, I. (1993). Population dynamics of animals in upredictably-changing tropical environments. Journal of Bioscience, 18, 425-455.	Search 1	Journal Article	Systematic observation	Indonesia	*Tetragonula minangkabau*	Beetles (*Procoryphaeus wallacei*)

Article	Source[1]	Publication Type	Study Type[2]	Study Location	Bee Species Studied	Pest or Disease Studied[3]
Ivorra, T., Hauser, M., Low, V. L., Tomberlin, J. K., Aliah, N. A. N., Cammack, J. A., & Heo, C. C. (2020). Hermetia illucens and Hermetia fenestrata (Diptera: Stratiomyidae) Colonization of "Spoiled" Stingless Bee Geniotrigona thoracica (Hymenoptera: Apidae) Hives in Malaysia. Insects, 11(11). https://doi.org/10.3390/insects11110737	Search 1	Journal Article	Case Study	Malaysia	*Geniotrigona thoracica*	Soldier Flies (*Hermetia illucens, Hermetia fenestrate*)
Krishnan, K. T., Neumann, P., Ahmad, A. H., & Pimid, M. (2014). A scientific note on the association of Haptoncus luteolus (Coleoptera: Nitidulidae) with colonies of multiple stingless bee species. Apidologie, 46(2), 262-264. https://doi.org/10.1007/s13592-014-0312-3	Search 1	Journal Article	Case Study	Malaysia	*Trigona thoracica, Heterotrigona itama, Tetragonula laeviceps*	Beetles (*Haptoncus luteolus*)
Lau, I. H., Hereward, J. P., Smith, T. J., Heard, T. A., & Walter, G. H. (2022). Inter-colony fights in Tetragonula stingless bees result in temporary mixed-species worker cohorts. *Apidologie*, *53*(4), 37.	Search 15	Journal Article	Case Study	Australia	*Tetragonula carbonaria*	Usurpation by other bees (con-specifics and *Tetragonula hockingsi*)
Loriga Pena, W., Fonte Carballo, L., & Demedio Lorenzo, J. (2014). Reporte de Aethina tumida Murray (Coleoptera, Nitidulidae) en colonias de la abeja sin aguijón Melipona beecheii Bennett de Matanzas y Mayabeque. Revista de Salud Animal, 36(3), 201-204.	Search 3	Journal Article	Systematic observation	Cuba	*Melipona beecheii*	Small Hive Beetle (*Aethina tumida*)
Macias-Macias, J. O., & Otero-Colina, G. (2004). Infestation of Pyemotes tritici (Acari: Pyemotidae) ON Melipona colimana (Hymenoptera: Apidae: Meliponinae): ACase Study. AGROCIENCIA, 38(5).	Search 1	Journal Article	Case Study	Mexico	*Melipona colimana*	Mites (*Pyemotes tritici*)
Macias-Macias, J. O., Tapia-Rivera, J. C., De la Mora, A., Tapia-Gonzalez, J. M., Contreras-Escareno, F., Petukhova, T., Morfin, N., & Guzman-Novoa, E. (2020). Nosema ceranae causes cellular	Reference Search	Journal Article	Systematic Manipulation	Mexico	*Melipona colimana*	Nosema

Article	Source[1]	Publication Type	Study Type[2]	Study Location	Bee Species Studied	Pest or Disease Studied[3]
immunosuppression and interacts with thiamethoxam to increase mortality in the stingless bee Melipona colimana. Sci Rep, 10(1), 17021. https://doi.org/10.1038/s41598-020-74209-3						
Maia-Silva, C., Hrncir, M., Koedam, D., Machado, R. J., & Imperatriz-Fonseca, V. L. (2013). Out with the garbage: the parasitic strategy of the mantisfly Plega hagenella mass-infesting colonies of the eusocial bee Melipona subnitida in northeastern Brazil. Naturwissenschaften, 100(1), 101-105. https://doi.org/10.1007/s00114-012-0994-1	Search 1	Journal Article	Systematic manipulation	Brazil	*Melipona subnitida*	Mantisflies (*Plega hagenella*)
Martínez, P. A., Alvarez, L. J., Garrido, P. M., Porrini, D. P., Muller, P. F., Alberoni, D., & Porrini, M. P. (2023). First record of Leptus spp. (Acari: Erythraeidae) parasitizing stingless bees (Apidae: Meliponini). Journal of Apicultural Research, 63(2), 367-372. https://doi.org/10.1080/00218839.2023.2244719	Search 1	Journal Article	Systematic observation	Argentina	*Schwarziana quadripunctata, Trigona spinipes, Melipona torrida, Scaptotrigona depilis*	Mites (*Leptus* spp.)
Martins de Oliveira, A. P., Venturieri, G. C., & Leon Contrera, F. A. (2013). Body size variation, abundance and control techniques of *Pseudohypocera kerteszi*, a plague of stingless bee keeping Bulletin of Insectology, 66, 203-208.	Search 1	Journal Article	Systematic manipulation	Brazil	*Melipona fasciculata, Melipona seminigra*	Phorid Flies (*Pseudohypocera kerteszi*)
Mascena, V. M., Nogueira, D. S., Silva, C. M., & Freitas, B. M. (2017). First record of the stingless bee Lestrimelitta rufa (Friese)(Hymenoptera: Apidae: Meliponini) in NE Brazil and its cleptobiotic behavior. *Sociobiology*, *64*(3), 359-362.	Search 15	Journal Article	Case Study	Brazil	*Melipona quinquefasciata*	Obligate cleptoparasitic stingless bee (*Lestrimelitta rufa*)
Meneses, H. M., Rocha, E. E. d. M., Eleutério, P., Mota, A. A., Lima-Verde, L. W., Dierings, A. C., & Freitas, B. M. (2024). First record of Hololepta (Leionota) reichii (Marseul, 1853) (Coleoptera: Histeridae) in meliponaries of the state of Ceará (Brazil) and notes on the methods used to control	Reference Search	Journal Article	Case Study	Brazil	*Melipona flavolineata, Melipona subnitida, Melipona fasciculat, Scaptotrigona*	Beetles (*Hololepta reichii*)

Article	Source[1]	Publication Type	Study Type[2]	Study Location	Bee Species Studied	Pest or Disease Studied[3]
this pest. Entomological Communications, 6. https://doi.org/10.37486/2675-1305.ec06030					*depilis, Frieseomelitta varia*	
Menezes, C., Coletto-Silva, A., Gazeta, G. S., & Kerr, W. E. (2009). Infestation by Pyemotes tritici (Acari, Pyemotidae) causes death of stingless bee colonies (Hymenoptera: Meliponina). Genet Mol Res, 8(2), 630-634. https://doi.org/10.4238/vol8-2kerr021	Reference Search	Journal Article	Case Study	Brazil	*Frieseomelitta varia, Tetragonula angustula*	Mites (*Pyemotes tritici*)
Michener, C. D. (1946). Notes on the habits of some Panamanian stingless bees (Hymenoptera, Apidae). *Journal of the New York Entomological Society*, *54*(3), 179-197.	Reference search	Journal Article	Case Study	Panama	*Trigona testaceicornis perilampoi*	Obligate cleptoparasitic bees (*Lestrimelitta limao*)
Moretto, G. (2000). Treatment against the forid fly Pseudohypocera kerteszi in Melipona quadrifasciata Lep Acta Scientiarum, 22(3), 651-653.	Reference Search	Journal Article	Systematic manipulation	Brazil	*Melipona quadrifasciata*	Phorid Flies (*Pseudohypocera kerteszi*)
Morfin, N., Gashout, H. A., Macías-Macías, J. O., De la Mora, A., Tapia-Rivera, J. C., Tapia-González, J. M., Contreras-Escareño, F., & Guzman-Novoa, E. (2020). Detection, replication and quantification of deformed wing virus-A, deformed wing virus-B, and black queen cell virus in the endemic stingless bee, Melipona colimana, from Jalisco, Mexico. International Journal of Tropical Insect Science, 41(2), 1285-1292. https://doi.org/10.1007/s42690-020-00320-7	Search 2	Journal Article	Systematic observation	Mexico	*Melipona colimana*	DWV, BQCV
Nacko, S., Hall, M., Duncan, M., Cook, J., Riegler, M., & Spooner-Hart, R. (2020). Scientific note on small hive beetle infestation of stingless bee (Tetragonula carbonaria) colony following a heat wave. Apidologie, 51(6), 1199-1201. https://doi.org/10.1007/s13592-020-00799-6	Search 1	Journal Article	Case Study	Australia	*Tetragonulla carbonaria*	Small Hive Beetle (*Aethina tumida*)
Neumann, P., Hoffmann, D., Duncan, M., Spooner-Hart, R., & Pettis, J. S. (2012). Long-range dispersal of small hive beetles. Journal of Apicultural Research,	Search 3	Journal Article	Systematic manipulation	Australia	*Tetragonula carbonaria*	Small Hive Beetle (*Aethina tumida*)

Article	Source[1]	Publication Type	Study Type[2]	Study Location	Bee Species Studied	Pest or Disease Studied[3]
51(2), 214-215. https://doi.org/10.3896/ibra.1.51.2.11						
Nkoba, K., Nelly, N. u., Pozo, M. I., Lattorff, H. M. G., Jaramillo, J., & Hundt, B. (2024). Black queen cell virus detected in endemic African stingless bees (Apidae: Meliponinae). International Journal of Tropical Insect Science, 44(5), 2353-2359. https://doi.org/10.1007/s42690-024-01303-8	Search 1	Journal Article	Systematic observation	Kenya	*Hypotrigona gribodoi, Meliponula bocandei, Meliponula ferruginea, Meliponula togoensis, Meliponula beccarii, Meliponula lendiliana, Dactylurina Schmidt, Liotrigona sp. Plebeina hildebranti*	Nosema, DWV, BQCV, APPV, CBPV, SBV, IBPV, KBV
Nunes-Silva, P., Piot, N., Meeus, I., Blochtein, B., & Smagghe, G. (2016). Absence of Leishmaniinae and Nosematidae in stingless bees. Sci Rep, 6, 32547. https://doi.org/10.1038/srep32547	Search 2	Journal Article	Systematic observation	Brazil	*Plebeia emerina, Tetragonisca fiebrigi*	Nosema, Protozoan parasites (Neogregarinorida, *Leishmania* spp.)
Oliveira França, S., Mascena Peixoto, C., Correia-Oliveira, M. E., Freitas de Melo, L., Melo Poderoso, J. C., Albany Costa, J., Pereira de Carvalho Costa, M. A., & Lopes de Carvalho, C. A. (2024). Viruses in Brazilian stingless bees. Journal of Apicultural Research, 63(2), 260-266. https://doi.org/10.1080/00218839.2024.2317066	Search 2	Journal Article	Systematic observation	Brazil	*Melipona scutellaris, Melipona quadrifasciata, Nannotrigona testaceicornis, Tetragonisca angustula, Scaptotrigona xanthotricha, Partamona helleri, Trigona spinipes*	DWV, BQCV, APPV, SBV, CBPV, IBPV, KBV

Article	Source[1]	Publication Type	Study Type[2]	Study Location	Bee Species Studied	Pest or Disease Studied[3]
Pereira, S. N., Alves, L. H. S., Costa, R. F. R. d., Prezoto, F., & Teixeira, E. W. (2021). Occurrence of the small hive beetle (Aethina tumida) in Melipona rufiventris colonies in Brazil. Sociobiology, 68(1). https://doi.org/10.13102/sociobiology.v68i1.6021	Search 1	Journal Article	Case Study	Brazil	*Melipona rufiventris*	Small Hive Beetle (*Aethina tumida*)
Porrini, M. P., Porrini, L. P., Garrido, P. M., de Melo, E. S. N. C., Porrini, D. P., Muller, F., Nunez, L. A., Alvarez, L., Iriarte, P. F., & Eguaras, M. J. (2017). Nosema ceranae in South American Native Stingless Bees and Social Wasp. Microb Ecol, 74(4), 761-764. https://doi.org/10.1007/s00248-017-0975-1	Search 1	Journal Article	Systematic observation	Argentina, Brazil	*Tetragonisca fiebrigi, Scaptotrigona jujuyensis, Tetragonisca angustula, Melipona fasciculata, Melipona quadrifasciata anthidioides, Melipona marginata, Melipona rufiventris, Melipona mandacaia*	Nosema
Purkiss, T., & Lach, L. (2019). Pathogen spillover from Apis mellifera to a stingless bee. Proc Biol Sci, 286(1908), 20191071. https://doi.org/10.1098/rspb.2019.1071	Search 1	Journal Article	Systematic manipulation	Australia	*Tetragonula hockingsi*	Nosema
Rech, A. R., Schwade, M. A., & Schwade, M. R. M. (2013). Abelhas-sem-ferrão amazônicas defendem meliponários contra saques de outras abelhas. *Acta Amazonica*, *43*, 389-393.	Reference Search	Journal Article	Case Study	Brazil	*Scaptotrigona* sp., *Duckeola ghilianii*, *Melipona fulva*	Obligate cleptoparasitic stingless bees *Lestrimelitta rufipes*
Robroek, B. J. M., de Jong, H., Arce, H., & Sommeijer, M. (2003). The development of Pseudohypocera kerteszi (Diptera, Phoridae), a kleptoparasite in nests of stingless bees (Hymenoptera, Apidae) in Central America. Proceedings of the Section Experimental	Reference Search	Journal Article	Systematic observation	El Salvador, Costa Rica	*Melipona beecheii*	Phorid Flies (*Pseudohypocera Kertesz*)

**Article**	**Source**[1]	**Publication Type**	**Study Type**[2]	**Study Location**	**Bee Species Studied**	**Pest or Disease Studied**[3]
and Applied Entomology - Netherlands Entomological Society, 14, 71-74.						
Roy, B. (2020). *Assessing pathogen risks to Australian stingless bees* Western Sydney University	Reference Search	Thesis	Systematic manipulation	Australia	*Tetragonula carbonaria, Tetragonula hockingsi, Austropledia australis*	Nosema, Bacterial Brood Disease (Shanks Brood Disease), BQCV
Sakagami, S. F., Roubik, D. W., & Zucchi, R. (1993). Ethology of the robber stingless bee, Lestrimelitta limao (Hymenoptera: Apidae). Sociobiology, 21(3).	Search 15	Journal Article	Systematic observation	Brazil, Panama	17 stingless bee species	Obligate cleptoparasitic stingless bees (*Lestrimelitta limao*)
Salt, G. (1929). A Contribution to the Ethology of the Meliponinae. Transactions of the Royal Entomological Society of London, 77(2), 431-470. https://doi.org/10.1111/j.1365-2311.1929.tb00693.x	Reference Search	Journal Article	Systematic observation	Brazil, Colombia	*Trigona Amalthea, Trigona capitata*	Phorid Flies (*Pseudohypocera nigrofascipes*)
Sanchez-Quilindo, B. A., Pizo-Barona, H. L., & Benavides Montano, J. A. (2024). Leptus alberti n. sp. (Trombidiformes: Erythraeidae) parasitizing free-living colonies of Apis mellifera, Partamona peckolti, Paratrigona eutaeniata and Tetragonisca angustula in Totoro and Valle del Cauca, Colombia. PLOS ONE, 19(12), e0311409. https://doi.org/10.1371/journal.pone.0311409	Search 1	Journal Article	Systematic observation	Colombia	*Partamona peckolti, Paratrigona eutaeniata, Tetragonisca angustula*	Mites (*Leptus alberti*)
Santos Louzado das Neves, V., Mascena Peixoto, C., Oliveira França, S., Correia-Oliveira, M. E., & Lopes de Carvalho, C. A. (2025). First record of Leptus sp. larvae on Melipona scutellaris. Journal of Apicultural Research, 1-6. https://doi.org/10.1080/00218839.2025.2504812	Reference Search	Journal Article	Systematic observation	Brazil	*Melipona scutellaris*	Mites (*Leptus* spp.)

Article	Source[1]	Publication Type	Study Type[2]	Study Location	Bee Species Studied	Pest or Disease Studied[3]
Segers, F. H., von Zuben, L., & Grüter, C. (2016). Local differences in parasitism and competition shape defensive investment in a polymorphic eusocial bee. *Ecology*, *97*(2), 417-426.	Search 15	Journal Article	Systematic observation and Systematic manipulation	Brazil	*Tetragonisca angustula*.	Obligate cleptoparasitic stingless bees (*Lestrimelitta limao*)
Shanks, J. L., Haigh, A. M., Riegler, M., & Spooner-Hart, R. N. (2017). First confirmed report of a bacterial brood disease in stingless bees. J Invertebr Pathol, 144, 7-10. https://doi.org/10.1016/j.jip.2017.01.004	Search 2	Journal Article	Systematic manipulation	Australia	*Tetragonula carbonaria*	Bacterial Brood Disease (Shanks Brood Disease)
Simoes, D., Bego, L. R., Zucchi, R., & Sakagami, S. F. (1980). Melaloncha sinistra Bogmeier, an endoparasitic phorid fly attacking Nannotrigona (Scaptotrigona) postica Latreille (Hymenoptera, Meliponinae). Review of Brazilian Entomology, 24(2), 137-142.	Reference Search	Journal Article	Case Study	Brazil	*Scaptotrigona postica*	Phorid Flies (*Melaloncha sinistra*)
Tapia-Gonzalez, J. M., Morfin, N., Macias-Macias, J. O., De la Mora, A., Tapia-Rivera, J. C., Ayala, R., Contreras-Escareno, F., Gashout, H. A., & Guzman-Novoa, E. (2019). Evidence of presence and replication of honey bee viruses among wild bee pollinators in subtropical environments. J Invertebr Pathol, 168, 107256. https://doi.org/10.1016/j.jip.2019.107256	Search 2	Journal Article	Systematic observation	Mexico	*Melipona colimana, Trigona fulviventris, Nannotrigona perilapoides, Scaptotrigona Mexicana*	DWV, BQCV, APPV, SBV, CBPV, IBPV
Teixeira, E. W., Ferreira, E. A., Luz, C., Martins, M. F., Ramos, T. A., & Lourenco, A. P. (2020). European Foulbrood in stingless bees (Apidae: Meliponini) in Brazil: Old disease, renewed threat. J Invertebr Pathol, 172, 107357. https://doi.org/10.1016/j.jip.2020.107357	Search 2	Journal Article	Systematic observation	Brazil	*Melipona marginata, Melipona mandacaia, Melipona quadrifasciata, Melipona compressipes, Melipona rufiventris, Melipona mondury*	DWV, BQCV, APPV, CBPV, IBPV, KBV, Nosema, Bacterial Brood Disease (European Foulbrood)

Article	Source[1]	Publication Type	Study Type[2]	Study Location	Bee Species Studied	Pest or Disease Studied[3]
Toledo-Hernandez, E., Moraga-Cáceres, E. U., Lormendez, C. C., Alvear-García, A., & Peña-Chora, G. (2021). First Report in North America of the Parasitic Small Hive Beetle, Aethina tumida, Infesting a Hive of Stingless Bee, Plebeia frontalis. SOUTHWESTERN ENTOMOLOGIST, 46(4).	Search 1	Journal Article	Case Study	Mexico	*Melipona beecheii*	Small Hive Beetle (*Aethina tumida*)
Ueira-Vieira, C., Almeida, L. O., de Almeida, F. C., Amaral, I. M. R., Brandeburgo, M. A. M., & Bonetti, A. M. (2015). Scientific note on the first molecular detection of the acute bee paralysis virus in Brazilian stingless bees. Apidologie, 46(5), 628-630. https://doi.org/10.1007/s13592-015-0353-2	Search 2	Journal Article	Systematic observation	Brazil	*Melipona scutellaris*	DWV, BQCV, APPV, SBV, CBPV, IBPV, KBV
Vijayakumar, K., Muthuraman, M., & Jayaray, R. (2013). Infestation of Carpoglyphus lactis (Linnaeus) (Acari: Carpoglyphidae) on Trigona iridipennis (Apidae: Meliponinae) from India. Scholarly Journal of Agricultural Science, 3(1), 25-28.	Reference Search	Journal Article	Case Study	India	*Trigona iridipennis*	Mites (*Carpoglyphus lactis*)
Xia, E., Keir, M., Tarlinton, B., Hauxwell, C., Buchmann, G., Lim, J., ... & Gloag, R. (2025). Queen turnover, nest usurpation and colony mortality in wild nests of the stingless bees Tetragonula carbonaria and Tetragonula hockingsi (Hymenoptera: Apidae). *Austral Entomology*, *64*(3), e70014.	Search 15	Journal Article	Systematic observation	Australia	*Tetragonula hockingsi, Tetragonula carbonaria*	Usurpation by other bees (*Tetragonula hockingsi*)
Zhang, L., Deng, Y., Zhao, H., Zhang, M., & Hou, C. (2021). Occurrence and Phylogenetic Analysis of DWV in Stingless Bee (Apidae sp.) in China: A Case Report. Front Insect Sci, 1, 748074. https://doi.org/10.3389/finsc.2021.748074	Search 2	Journal Article	Systematic observation	China	*Tetragonula laeviceps*	DWV, BQCV, APPV, SBV, CBPV, IBPV, Apis Mellifers Filamentous Virus

1- For studies located through database searches, source is the first search in which the study was found (Table 1, main text). All studies found via database searches were first found in the initial English language searches (i.e. searches in Spanish and

Portuguese did not yield any studies suitable for inclusion that were not found in the English search). “Reference Search” indicates that a study was located within the reference list of another study found through the database searches.

2- “Systematic manipulation” studies report on work where researchers manipulated test organisms or the environment to answer a specific question.
“Systematic observation” studies report on findings where researchers observed organisms without artificial manipulation to answer a specific question.
“Case studies” report on observations of an organism or phenomenon without researchers setting out to answer a specific question. They are typically studies undertaken after researchers became aware of a specific event in progress.

3- Abbreviations used are as follow: DWV – Deformed Wing Virus, BQCV – Black Queen Cell Virus, ABPV- Acute Bee Paralysis Virus, CBPV – Chronic Bee Paralysis Virus, SBV - Sacbrood Virus, IBPV – Israeli Bee Paralysis Virus, KBV – Kashmiri Bee Virus

S3 – Initial Search Results

Table S3 compares the number of English language search results found prior to screening in the stingless bee related and honeybee related searches. Honeybee related searches were not subjected to screening; this search was intended simply to gain an indication of the differing number of search results found for the two groups of bees.

**Table S3**			
**Search Number**		**Results - Stingless Bees**	**Results – Honey Bees**
**1**	pest OR parasit* OR predat*	273	5727
**2**	diseas* OR virus* OR pathogen* OR bacter* OR fung*	499	8972
**3**	"small hive beetle" OR "aethina tumida"	24	339
**4**	phorid OR phoridae OR Dohrniphora	13	27
**5**	Ceriania OR syrph* OR hoverfl* OR "hover fly" OR "hover flies"	26	429
**6**	Syntretus	1	0
**7**	"wax moth" OR "wax moths" OR Galleria OR Achroia	3	203
**8**	Nosema	25	1558
**9**	foulbrood OR "foul brood"	10	945
**10**	Hyg*	32	737
**11**	mite* OR acari	27	3103
**12**	wasp* OR bembix	140	1650
**13**	hermetia OR "soldier fly" OR "soldier flies"	3	24
**14**	Mantispid* OR mantisfly OR mantisflies OR “mantis flies” OR “mantis flies”	2	1
**15**	cleptoparas* OR kleptoparas* OR cleptobios* OR kleptobios* OR robb* OR usurp* OR Lestrimelitta OR Cleptotrigona	106	310
